# The regeneration-responsive element *careg* monitors activation of Müller glia after MNU-induced damage of photoreceptors in the zebrafish retina

**DOI:** 10.3389/fnmol.2023.1160707

**Published:** 2023-04-17

**Authors:** Thomas Bise, Catherine Pfefferli, Marylène Bonvin, Lea Taylor, Heidi E. L. Lischer, Rémy Bruggmann, Anna Jaźwińska

**Affiliations:** ^1^Department of Biology, University of Fribourg, Fribourg, Switzerland; ^2^Interfaculty Bioinformatics Unit, University of Bern, Bern, Switzerland; ^3^Swiss Institute of Bioinformatics (SIB), Lausanne, Switzerland

**Keywords:** regeneration enhancer, retina regeneration, MNU-injury, rods, cones, Müller glia, TOR, rapamycin

## Abstract

In contrast to mammals, zebrafish can regenerate their damaged photoreceptors. This capacity depends on the intrinsic plasticity of Müller glia (MG). Here, we identified that the transgenic reporter *careg*, a marker of regenerating fin and heart, also participates in retina restoration in zebrafish. After methylnitrosourea (MNU) treatment, the retina became deteriorated and contained damaged cell types including rods, UV-sensitive cones and the outer plexiform layer. This phenotype was associated with the induction of *careg* expression in a subset of MG until the reconstruction of the photoreceptor synaptic layer. Single-cell RNA sequencing (scRNAseq) analysis of regenerating retinas revealed a population of immature rods, defined by high expression of *rhodopsin* and the ciliogenesis gene *meig1*, but low expression of phototransduction genes. Furthermore, cones displayed deregulation of metabolic and visual perception genes in response to retina injury. Comparison between *careg:EGFP* expressing and non-expressing MG demonstrated that these two subpopulations are characterized by distinct molecular signatures, suggesting their heterogenous responsiveness to the regenerative program. Dynamics of ribosomal protein S6 phosphorylation showed that TOR signaling became progressively switched from MG to progenitors. Inhibition of TOR with rapamycin reduced the cell cycle activity, but neither affected *careg:EGFP* expression in MG, nor prevented restoration of the retina structure. This indicates that MG reprogramming, and progenitor cell proliferation might be regulated by distinct mechanisms. In conclusion, the *careg* reporter detects activated MG, and provides a common marker of regeneration-competent cells in diverse zebrafish organs, including the retina.

## Introduction

The visual system is fundamentally similar in zebrafish and humans. In both these species, the retina is composed of three nuclear and two plexiform layers (Chhetri et al., 2014; Niklaus and Neuhauss, 2017; Zang and Neuhauss, 2021). Despite the comparable structure, the loss of photoreceptor cells, rods, and cones, is irreversible in mammals, whereas they can be fully replaced in zebrafish (Wan and Goldman, 2016; Angueyra and Kindt, 2018). In this species, the subsequent impairment to visual function is transient and progressively returns to the original level (Hammer et al., 2021; Barrett et al., 2022). Beside the retina, other complex organs, such as fins, heart, spinal cord, and brain, can be fully restored in adult zebrafish (Gemberling et al., 2013; Sehring et al., 2016; Mokalled and Poss, 2018; Marques et al., 2019). This capacity mostly relies on the conversion of quiescent functional cells into proliferative precursors. This intrinsic plasticity, which accounts for regenerative organogenesis and neurogenesis, remains insufficiently understood at the molecular level.

Zebrafish can cope with damaged photoreceptors by increasing cellular survival programs or by replacing injured cells with new successors (Wan and Goldman, 2016). To study renewal of rods and cones in the adult zebrafish retina, several injury models have been established, such as genetic cells ablation, phototoxic light exposure, mechanical puncture, and chemical injuries (Senut et al., 2004; Thomas and Thummel, 2013; DiCicco et al., 2014; Oel et al., 2015; Hanovice et al., 2019; Kramer et al., 2021; Cocchiaro et al., 2022; Iribarne and Hyde, 2022). Exposure to methylnitrosourea (MNU) has been used to model diseases that result in primary photoreceptor cell death, in both zebrafish and rodents (Tsubura et al., 2010; Tappeiner et al., 2013). A chemical intervention is particularly advantageous as a robust and non-surgical injury model causes extensive damage to photoreceptors in the adult zebrafish retina (Tappeiner et al., 2013). One hour exposure to MNU is sufficient to induce apoptotic death, predominantly in rods. However, other possible aberrations have not been deeply investigated. The integrative analysis of cellular and molecular changes in rods and cones at the level of protein localization and gene expression is essential to understand the plasticity of these neuroepithelial cells upon chemical stress.

After a lesion, the zebrafish can regenerate lost photoreceptors *via* activation of an eye-specific glia, called Müller glia (MG) (Vihtelic and Hyde, 2000; Goldman, 2014). These cells possess thin cytoplasmic processes that span the entire thickness of the retina. Activated MG can change their gene expression profile and undergo asymmetrical cell division, producing an undifferentiated progenitor cell (Nagashima et al., 2013). These progenitors further symmetrically proliferate to amplify their numbers, and thereafter undergo interkinetic nuclear migration, followed by differentiation into photoreceptors or other neuronal cells (Goldman, 2014; Lahne and Hyde, 2016; Lahne et al., 2021). The step of MG reprogramming into multi-potent neuronal progenitors is a distinctive feature of the zebrafish retina. Fluorescent reporters of MG, such as *gfap:EGFP* or *olig2:EGFP*, have facilitated retina regeneration studies (Thummel et al., 2008; Campbell and Hyde, 2017). Thus, transgenic zebrafish strains could serve to investigate transcriptional dynamics of cellular conversions.

**FIGURE 1:**
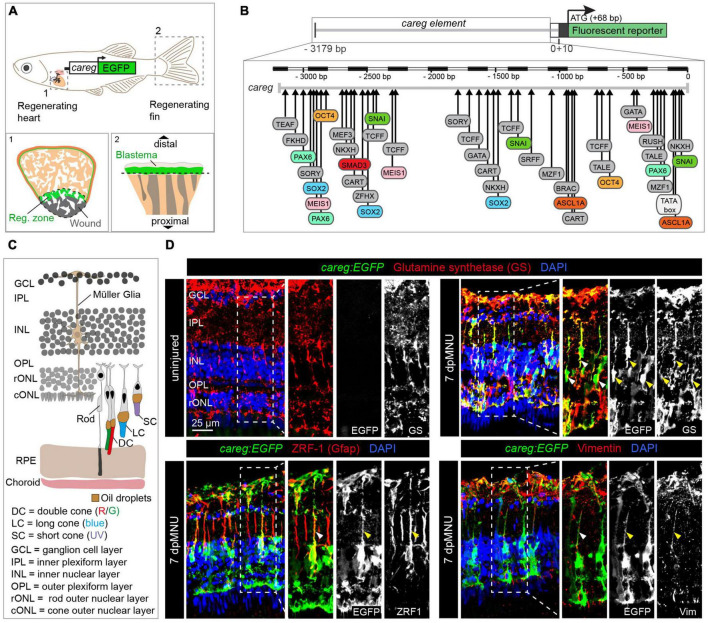
The *careg* regulatory element is activated in Müller glia during retina regeneration. **(A)** Schematic representation of the transgenic zebrafish line carrying the *careg* regulatory element upstream of the *EGFP* reporter, based on Pfefferli and Jaźwińska (2017). The *careg:EGFP* transgene is activated during heart (1) and fin (2) regeneration. **(B)** Prediction of transcription factor binding sites in the *careg* sequence with MatInspector (Genomatix) (Pfefferli and Jaźwińska, 2017). **(C)** Schematic representation of the retina in adult zebrafish, with an illustration of photoreceptors, based on Raymond and Barthel (2004) and Lagman et al. (2015). Outer segments of cones are colored according to their spectral sensitivity. Abbreviations are listed on legend at the bottom of the drawing. **(D)** Immunofluorescence stained sections of uninjured and 7 days post-MNU treatment (dpMNU) retinas of *careg:EGFP* (green) transgenic fish using three Müller glia markers (red): glutamine synthetase (GS), vimentin and the glial fibrillary acidic protein (GFAP, visualized with ZRF-1 antibodies). Nuclei are stained with DAPI (blue). In uninjured retina, no *careg:EGFP* is detected. At 7 dpMNU, *careg:EGFP* partially overlaps with some cells expressing GS, GFAP, and vimentin (arrowheads). In this, and all subsequent figures, a dashed frame demarcates the magnified area shown in adjacent panels. *N* ≥ 3 (number of fish).

Non-coding elements, either promoters or enhancers, provide valuable biosensors for the switch from quiescence to mobilization in regeneration of various organs (Rodriguez and Kang, 2020; Suzuki and Ochi, 2020). Despite the diversity of tissue types, injury-induced activation has been shown to involve common regulatory mechanisms (Kang et al., 2016; Yuan et al., 2018; Goldman and Poss, 2020). In agreement with the existence of regeneration-responsive genetic sensors, we have previously identified that a non-coding element, called *careg*, is transiently upregulated in regeneration-participating cells of the fin and the heart, despite the structural differences between both organs (Figure 1A; Pfefferli and Jaźwińska, 2017). The *careg* element contains a 3.18 kb sequence of the *connective tissue growth factor a* (*ctgfa*) promoter (Figure 1B; Chiou et al., 2006). While the *careg* element can be used to monitor the regenerative activation of fin mesenchyme and ventricular cardiomyocytes, its properties as a regeneration-responsive reporter have not yet been explored in neural tissues, such as the retina.

Here, we identified that *careg:EGFP* is a biosensor of regeneration-participating cells in the retina. Thus, this study together with our previous report demonstrates that *careg* is a common reporter of regeneration-participating cells in diverse organs. It is not detected in the uninjured retina but becomes temporarily induced in MG after MNU chemical injury. To assess whether *careg:EGFP* expression is associated with photoreceptor damage, we employed immunofluorescence analysis to examine rods and cones. To elucidate the molecular signature of regeneration-participating cells, we conducted single-cell RNA sequencing (scRNAseq) analysis at three time-points after MNU treatment, and compared to uninjured controls. Gene expression profiles of rods, cones, and MG provided new insights into transcriptional changes of MNU-damaged photoreceptors and the restorative process. *careg:EGFP*-expression predominantly demarcated a subpopulation of activated MG. We also analyzed the dynamics of TOR signaling in *careg:EGFP* retinas. Inhibition of TOR signaling resulted in reduced proliferation, however, *careg* reporter expression and regeneration were unaffected. This study indicates that distinct mechanisms might be involved in activation of MG and progenitor cell proliferation during retina regeneration.

## Materials and methods

### Animal lines and procedures

The present work was performed with adult fish between 12 and 24 months old. Wild-type fish were AB (Oregon) and transgenic lines were *careg:EGFP*, originally named as *Tg(ctgfa:EGFP)^zf^*^620^ (also named *cnn2a:EGFP*; ZFIN Database ID: ZDB-ALT-160802-2) (Chiou et al., 2006), *Tg(careg:dmKO)^fri^*^1^ (ZFIN Database ID: ZDB-ALT-180626-1) (Pfefferli and Jaźwińska, 2017), *Tg(–3.5ubb:loxP-EGFP-loxP-mCherry)^cz^*^1701^, referred to as *ubi:EGFP* (also named *ubi:Switch*; ZFIN Database ID: ZDB-ALT-110124-1) (Mosimann et al., 2011), and *TgBAC(gfap-GFP)*^zf^*^167^* (ZFIN Database ID: ZDB-ALT-100308-3) (Lam et al., 2009).

The retina injury was induced by MNU treatment and it was performed following the previously established method (Maurer et al., 2014). Briefly, fish were treated for 1 h in 1 L of system water containing 150 mg of MNU. Following the treatments and procedures, fish were kept in 1 L tank apart from the system for 1 day, then reintegrated to the system during the period of regeneration. For scRNA-seq experiment, control fish were treated with inactivated MNU heated at 90°C for 30 min. For chemical inhibition experiments, the animals were pre-treated for 2 days with water containing 1 μM rapamycin (Selleckchem) before MNU-treatment, followed by 2, 7, or 22 days after MNU treatment. A total of 0.1% DMSO was used as control, as solvent for preparation of stock concentration of the drug.

Animal experimentation was performed in accordance with Swiss regulations and approved by the Cantonal Veterinary Office of Fribourg, Switzerland.

### Immunofluorescence and histology

At the end of experiments, the eyes were collected in daylight and fixed overnight at 4°C in 4% paraformaldehyde. They were then rinsed in PBS and equilibrated in 30% sucrose for a minimum of 3 h. Lenses were removed before embedding in Tissue-Tek OCT compound (Sakura Finetek Europe B.V.) and cryo-sectioned at a thickness of 16 μm. The immunofluorescence procedures were performed as previously described (Chablais et al., 2011). The following primary antibodies were used: mouse anti glutamine synthetase (GS) at 1:200 (MAB302; Millipore, Billerica, MA, USA), (provided by Enzmann V. Group, University of Bern), mouse anti-proliferating cell nuclear antigen (PCNA) at 1:200 (Clone PC10; M0879; DAKO), rabbit anti-MCM5 at 1:500 (kindly provided by Soojin Ryu, Heidelberg); mouse IgG ZRF-1/GFAP 1:100 (ZIRC, University of Oregon), mouse IgG ZPR-1 1:100 (ZIRC, University of Oregon), mouse IgM Xap-1 (Clone 3D2) at 1:10 (DSHB), mouse IgG3 Xap-2 (Clone 5B9) at 1:10 (DSHB), mouse anti Vimentin at 1:50 (40E-C, DSHB) Chick anti-GFP antibody at 1:500 (GFP-1020; Aves labs); Rabbit anti KO2 at 1:200 (PM051M, MBL International Corporation). The Alexa-Fluor conjugated secondary antibodies (Jackson Immunoresearch) were used at 1:500, and DAPI was used at 1:2,000.

Proliferating cell nuclear antigen staining required heat-induced epitope retrieval in 10 mM citric buffer, pH 6.0, 120°C for 3 min, whereas all other staining were performed without this step, according to standard immunofluorescence staining protocol (Bise et al., 2020).

### Retina dissection, dissociation, and single cell suspension for RNA-seq experiment

The fish were dark-adapted for 1 day prior to retina collection. Zebrafish eyeballs were collected on ice, in a darkroom under red light in brown Eppendorf 1.5 ml containing 1 ml ice-cold PBS1x. Under a binocular microscope in ice-cold PBS, each retina was directly extracted from eyeball, minced into small pieces, and placed in 500 μl of ice-cold RNAse-free PBS within 2 min following eyeball collection and for a maximum of 30 min (to avoid RPE sticking on the neuroepithelium).

To dissociate retinal cells, PBS was replaced with a solution of liberase 2.5 mg/ml (Merck, Liberase DH) at 35°C and incubated at 28°C for 20 min. To ensure maximal tissue dissociation, 3–4 rounds of trituration were done using wide bore pipet tips. The enzymatic reaction was stopped by adding 100 μl of 10% BSA.

The resulting cell suspension was then filtered using 40 μm Cell strainer (Corning) and centrifuged at 800 *g* for 5 min at 4°C. Supernatant was discarded and the pellet resuspended in 500 μl of PBS containing 0.5% BSA. Cell concentration and viability were determined using both MACSQuant flow cytometer and hemocytometer (improved Neubauer). Cell viability was determined using Propidium Iodide (PI) with both methods.

### 10X genomics and sequencing

Libraries for scRNA-seq were prepared using the Chromium Single Cell 3′ Library and Gel Bead Kit v3 (10X Genomics), according to the manufacturer’s protocol (User Guide). Briefly, wells were loaded after calculating single-cell suspension concentration of each sample equal to 1,200 cells/μl. Targeted recovery rate was approximately 10,000 cells. 10X Chromium Chip performed GEMs generation, reverse transcription, and cDNA amplification. Illumina NovaSeq 6000 S2 flow cell was used for deep sequencing generating paired-end reads. Different sequencing cycles were performed for the different reads, R1 and R2. R1, contained 10X barcodes and UMIs, in addition to an Illumina i7 index. R2, contained the transcript-specific sequences.

### 10x data processing

Illumina BCLs data were demultiplexed into FASTQ files using Cellranger mkfastq pipelines v3.0.2 according to the 10X genomic support. To allow EGFP transcripts detection, referenced and annotated genome GRCz11 from NCBI were manually modified to add EGFP sequence. Reads were aligned on referenced genome using Cellranger Count pipeline.

Using Seurat package from Satijas lab in R (v4.1.0) (Stuart et al., 2019), biological duplicates were merged into single conditions and filtered according to metadata. Genes with UMIs <250 and <200 were trimmed. The Scater tool was used to identify low-quality cells based on experiment-specific aspects of the data (McCarthy et al., 2017). This includes cells with a low library size, low number of expressed genes, high proportion of mitochondrial reads.

All datasets were then associated and merged into a single object for all further analysis. The data was normalized using a SCTransform normalization (Hafemeister and Satija, 2019), which builds regularized negative binomial models of gene expression in order to account for technical artifacts while preserving biological variance. During the normalization, we also removed confounding sources of variation (mitochondrial and ribosomal mapping percentage). The quality control report is included (Supplementary Data 1).

An integration analysis (dim = 30) corrected for confounders and batch effects (technical and biological variability) between samples. We identified single-cell clusters through k-nearest neighbors and a shared nearest neighbor modularity optimization. Clusters were determined with a resolution of 0.2 and visualized through UMAP dimensional reduction. Cell clusters were identified by specific marker genes according to previous published studies (Hoang et al., 2020; Ogawa and Corbo, 2021). Subsets of *careg:EGFP* expressing cells were obtained using the subset function in Seurat v4.1.0.

Genes upregulated in each cluster were identified using the FindAllMarkers function in Seurat v4.1.0 and are provided in Supplementary Table 1. Genes differentially expressed in specific cell types between post-MNU time-points (3, 7, and 10 dpMNU merged datasets) and uninjured control were obtained using the FindMarkers function in Seurat v4.1.0 using non-parametric Wilcoxon rank sum test. The DoHeatmap function of the Seurat package was used to create heatmaps of scaled gene expressions. GO term enrichment analysis were done using the topGO package (Alexa and Rahnenfuhrer, 2022), applying the Fisher’s exact test.

Additionally, all cells from clusters 1 and 9 were subset and reanalyzed following the approach above. Cell clusters were identified by specific marker genes and the new cluster 0–5 were merged into one cluster.

### Image analysis and quantification

Fluorescent images were taken with a Leica SPE II confocal microscope, and ImageJ 1.53f51 software was used for subsequent measurements. A minimum of 3 retinas were used per experiment and 2–3 representative images were taken per individual (*n*). From these, one representative image was selected for the relevant figure. Images of the same specimens were taken for area quantification. The area of *careg:EGFP* or p-rpS6 positive signal was measured using thresholds in ImageJ and was compared with the total area of the analyzed retina fragment.

Quantification of PCNA-positive nuclei was performed using the plugins colocalization and ITCN counter of ImageJ, and was normalized to the number of DAPI-positive nuclei. Error bars correspond to standard error of the mean (SEM). Significance was calculated using one-way ANOVA with Sidák multiple comparison test. Statistical analyses were performed with GraphPad Prism. All results are expressed as the mean ± SEM.

## Results

### The *careg* reporter is induced in Müller glia after MNU-mediated damage

To identify a transgenic tool for monitoring retina regeneration, we investigated the expression of a *cis*-regulatory DNA element, called *careg*, which we have previously characterized in the fin blastema and the peri-injured myocardium (Figure 1A; Pfefferli and Jaźwińska, 2017). Bioinformatic analysis of the *careg* sequence predicted various transcription factor-binding sites, many of which are known to regulate retina regeneration or development, such as *pax6, ascl1a, meis1*, and *oct4* (Bessa et al., 2008; Thummel et al., 2010; Gorsuch et al., 2017; Sharma et al., 2019; Figure 1B). Thus, the expression of this reporter might be regulated in the retina. Firstly, we aimed to investigate whether *careg* is expressed in the zebrafish eye during development. Between 2 and 5 days post-fertilization (dpf), *careg:EGFP* was detected in the optic nerve, whereas at 6 and 7 dpf, it was also observed in the retinal pigmented epithelium (RPE) and the corneal stroma (Supplementary Figure 1A). In adult zebrafish, the transgene expression expanded into the iris and certain blood vessels irrigating the eye. Importantly, no expression was observed in the adult retina (Supplementary Figure 1B). These analyses demonstrate that *careg:EGFP* is not expressed in retinal neurons, the germinal zone and glia cells at any stage.

The retina consists of several layers of specialized cells (Raymond and Barthel, 2004; Figure 1C). To assess the reactivity of the *careg:EGFP* reporter during retina regeneration, we aimed to damage the rod outer nuclear layer (rONL), which also comprises nuclei of UV cones. We applied a pulse treatment with MNU that causes disintegration of this layer after 5–8 days (Tappeiner et al., 2013). Our analysis of retina sections at 7 days post-MNU treatment (dpMNU) revealed that *careg:EGFP* was activated in elongated cells spanning all retinal layers, that resemble MG (Figure 1D). To identify these cells, we analyzed three MG markers, glutamine synthetase (GS), the glial fibrillary acidic protein (GFAP, visualized with ZRF-1 antibody), which is confined to a thin segment of MG, and the intermediate filament vimentin (Luna et al., 2010; Gao et al., 2021). We found that 62 ± 2% (*n* = 4) of *careg:EGFP*-positive area overlapped with GS staining and displayed a positional association with ZRF1- and vimentin-immunoreactive segments (Figure 1D). We concluded that at 7 dpMNU, the majority of *careg*-expressing cells are MG, which are known to have a regenerative potential in the zebrafish retina (Fischer and Reh, 2001).

To test the specificity of *careg* activation after retina injury, we used another transgenic line containing a fluorescent protein with a rapid turnover. Specifically, we selected destabilized monomeric Kusabira-Orange 2 (dmKO2), which contains a C-terminal PEST domain targeted for rapid degradation *via* ubiquitination (Collery and Link, 2011). Double transgenic fish, *careg:EGFP;careg:dmKO2* displayed a partially overlapping pattern of both reporters, whereby nearly all dmKO2-positive cells were also EGFP-positive (Supplementary Figures 2A, B). Furthermore, we used the fish strain *TgBAC(gfap-GFP)^zf^*^167^, which detects a subpopulation of MG (Lam et al., 2009) and generated double transgenic fish by crossing with *careg:dmKO2* (Supplementary Figure 2C). Consistent with previous studies (Conedera et al., 2017), *gfap:GFP^zf^*^167^ was detected mostly in the thin segment of MG often in close proximity to *careg:dmKO2*-positive cell (Supplementary Figure 2D). Taken together, we concluded that a population of *careg*-expressing cells belong to MG, which are activated during retina regeneration.

### The *careg* reporter monitors activated Müller glia throughout the entire regenerative process

**FIGURE 2:**
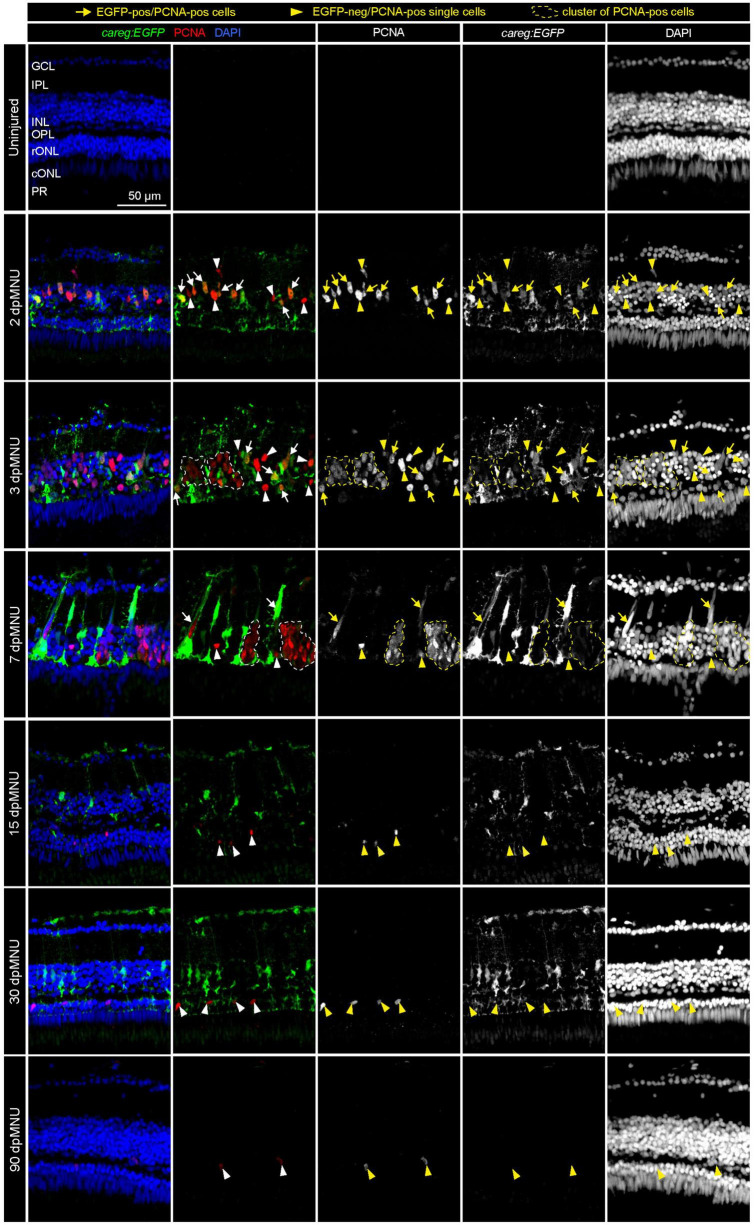
Proliferative cells of regenerating retinas include *careg:EGFP*-positive cells with an elongated nuclei. Sections of *careg:EGFP* (green) retinas, immunostained for the G1/S-phase cell cycle marker PCNA (red) and the nuclear marker DAPI (blue). Expression of *careg:EGFP* (green) is absent in the uninjured retina, but it is induced at 2 dpMNU and persists until 30 dpMNU. In the inner nuclear layer (INL), EGFP/PCNA double positive cells with an elongated nucleus (arrows) can be observed at 2, 3, and 7 dpMNU. Single EGFP-negative and PCNA-positive cells with roundish nuclei (arrowheads) are observed at all time-points after injury. Clusters of EGFP-negative and PCNA-positive cells (encircled with a dashed line) correspond to progenitor cells at 3 and 7 dpMNU. At 15 and 30 dpMNU, PCNA-positive cells are present in the outer nuclear layer (ONL). At 90 dpMNU, no EGFP-positive cells are detected. GCL, ganglion cell layer; IPL, inner plexiform layer; INL, inner nuclear layer; OPL, outer plexiform layer; rONL, rod outer nuclear layer; cONL, cone outer nuclear layer; PRL, photoreceptors layer. *N* ≥ 3 (number of fish).

Activated MG undergo an asymmetric cell division to give rise to a pool of progenitor cells (Nagashima et al., 2013). To investigate whether *careg:EGFP* was present in proliferative cells, we performed immunofluorescence analysis using G1/S phase markers, namely, PCNA and MCM5, at different time-points after injury. First, we found that the immunostaining pattern of both these antibodies was very similar, validating their specificity (Supplementary Figure 3). The earliest *careg:EGFP* expression started on day 1 after injury and became evident at 2 dpMNU (Figure 2 and Supplementary Figure 3). Thus, this reporter can function as an early transgenic sensor of tissue activation after damage. At 2 and 3 dpMNU, we observed a remarkable colocalization between *careg:EGFP^+^* cells and PCNA*^+^* or MCM5*^+^* nuclei within the inner nuclear layer (Figure 2 and Supplementary Figure 3), which comprise MG nuclei among other neurons (Figure 1C). On day 7, *careg:EGFP^+^* cells contained PCNA*^+^* or MCM5*^+^* nuclei, however, clusters of small proliferative cells were mostly GFP-negative. This suggests that *careg:EGFP* is predominantly expressed in activated MG, but not in the derived progenitors.

At 15 and 30 dpMNU, *careg:EGFP* remained in the inner nuclear layer, whereas a few proliferative cells were detected mostly in the outer nuclear layer; these cells were not demarcated by *careg:EGFP*. This indicates that the activation of this reporter persists throughout the entire regenerative process in activated MG, including a phase of declining cell proliferation. At 90 dpMNU, the fully regenerated retina contained no *careg:EGFP*^+^ cells, demonstrating a reversible regulation of the reporter in the repaired organ (Figure 2 and Supplementary Figure 3). Based on these findings, we concluded that *careg:EGFP* provides a unique transgenic tool for labeling activated MG from the primary to the terminal phase of regeneration.

### MNU-treatment damages rods and UV cones, leading to disruption of the outer plexiform layer

**FIGURE 3:**
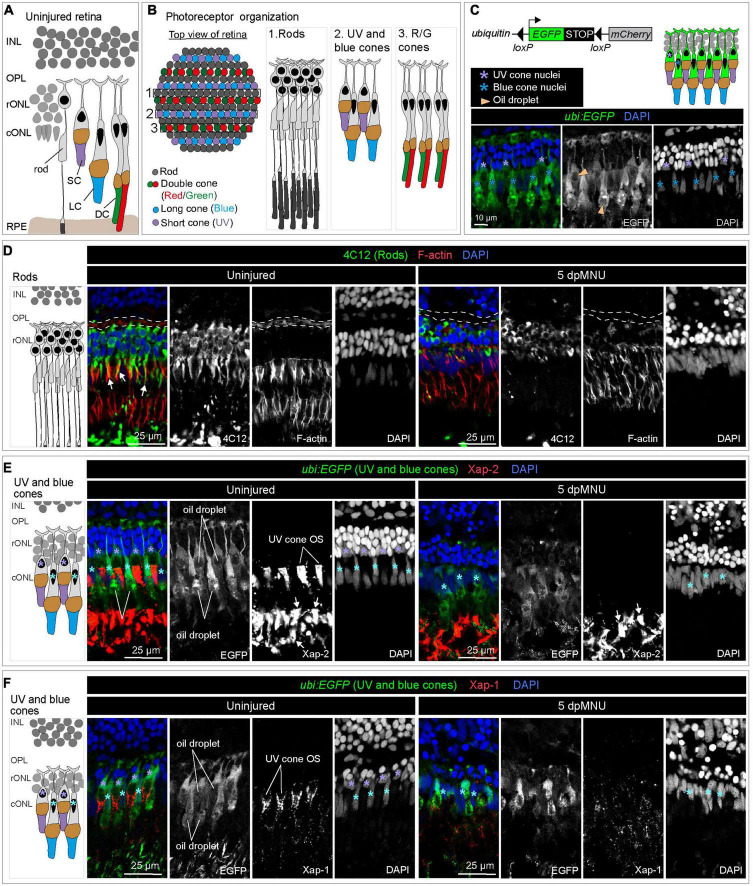
MNU-mediated injury predominantly affects rods, UV-cones and the outer plexiform layer. Schematics of photoreceptor organization in a transversal section **(A)** and frontal flattened view **(B)**, based on Raymond and Barthel (2004), Lagman et al. (2015), and Noel et al. (2021). INL, inner nuclear layer; rONL, rod outer nuclear layer; cONL, cone outer nuclear layer; OPL, outer plexiform layer; RPE, retinal pigmented epithelium; DC, double cones (red/green spectral sensitivity); LC, long single cones (blue spectral sensitivity); SC, short single cone (UV spectral sensitivity). **(C)** Ubiquitin-promoter driven *loxP-EGFP-loxP-mCherry* transgene is expressed in blue and UV cones. Top panel shows a schematic illustration and the bottom panel displays a section of uninjured retina with *ubi:EGFP* transgene expression (green) and DAPI (blue). **(D)** Transversal retinal section stained with 4C12 antibody to visualize cell bodies and inner segments of rods (green), Phalloidin to detect F-actin (red) and DAPI (blue). In uninjured retina, colocalization between 4C12 and Phalloidin (arrows) is detected at the level of inner segments. F-actin is also present in the photoreceptor processes in the outer plexiform layer (outlined with a dashed lines). At 5 dpMNU, F-actin in the outer plexiform layer is missing and rod cells bodies and their inner segments are disorganized. **(E)** Identification of Xap-2 antibody (red) as a marker of photoreceptor outer segments on section of *ubi:EGFP* transgenic retinas. A strong expression is detected distally to the oil droplet in the UV cones (purple asterisks) and blue cones (cyan asterisks). **(F)** Identification of Xap-1 antibody (red) as a marker of the cone outer segment on section of *ubi:EGFP* transgenic retinas. A dotty localization of Xap1 is enriched distally to the oil droplet in the UV cones (purple asterisks). Dots of Xap-1 labeling are also observed in the outer segments of other photoreceptors, but at much less concentrated level. *N* ≥ 3.

**FIGURE 4:**
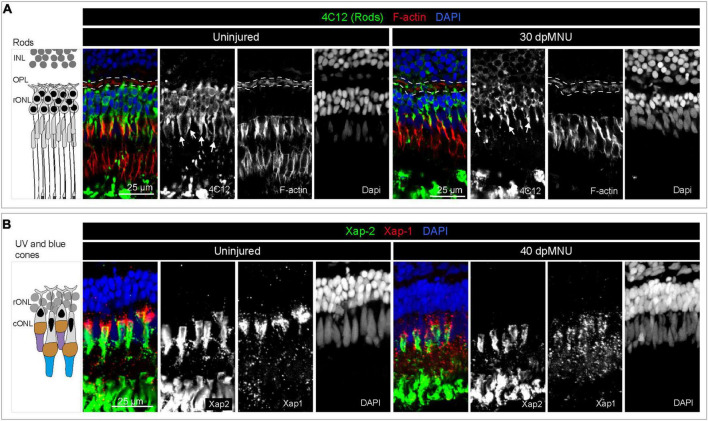
Restoration of rods and UV cones after MNU-injury. **(A)** Schematic illustration and sections of intact and 30 dpMNU retinas immunostained against the rod marker 4C12 (green), the F-actin marker Phalloidin (red) and DAPI (blue). The distribution of rods (4C12, green) and synaptic processes of the OPL (encircled with a dashed line) are restored at 30 dpMNU. **(B)** Schematic illustration and sections of intact and 40 dpMNU retinas immunostained with Xap-2 (green), Xap-1 (red) antibodies, and DAPI (blue). The outer segment of UV-cones is restored at 40 dpMNU. INL, inner nuclear layer; OPL, outer plexiform layer; rONL, rod outer nuclear layer; cONL, cone outer nuclear layer. *N* = 3.

Methylnitrosourea (MNU) treatment predominantly damages rods, as shown by the Tunel assay and quantification of cells in the outer nuclear layer (Tappeiner et al., 2013). However, the phenotypic characterization of the MNU-injured retina remains incomplete. To better understand which cellular changes may activate *careg:EGFP* upregulation in MG, we aimed to assess how MNU-injury disrupts photoreceptor organization (Figures 3A, B). We applied the 4C12 antibody that detects rod cell bodies, their inner segments, including myoid, and their outer segments (Sotolongo-Lopez et al., 2016). We combined this marker with Phalloidin, which labels filamentous actin (F-actin) of photoreceptor inner segments and the outer plexiform layer (Nadolski et al., 2020). At 5 dpMNU, 4C12-immunoreactive cell bodies of rods were present, but showed a contracted and disorganized appearance, whereas the myoid structure was no longer detectable (Figure 3D). Furthermore, no overlap between 4C12 and F-actin was apparent in the myoid of the inner segment, suggesting the loss of distinctive rod morphology (Figure 3D). In addition, Phalloidin staining of the outer plexiform layer was abolished after MNU-treatment, suggesting defects at the synaptic ends of photoreceptors and second level neurons. At 30 dpMNU, 4C12 and F-actin displayed a pattern comparable to the uninjured retina, suggesting regeneration (Figure 4A). These data demonstrate that MNU-treatment induces transient degenerative processes in rods, perturbing interconnections between photoreceptors and neurons.

In our attempts to perform lineage tracing analysis with the previously validated *careg:Cre-ERT2* driver (Pfefferli and Jaźwińska, 2017), we identified that the *ubi:loxP-EGFP-Stop-loxP-mCherry* transgene, here abbreviated as *ubi:EGFP*, is specifically expressed in UV and blue cones in the retina (Figure 3C). The *ubi:EGFP* was not activated in rods or ZPR-1-immunoreactive double red/green cones (Supplementary Figures 4A, B). Although CreERT2-*loxP*-mediated recombination was inefficient in the retina, as shown by negligible switching from EGFP to mCherry upon hydroxytamoxifen treatment, we identified that *ubi:EGFP* alone can serve as a transgenic tool to demarcate UV and blue cones.

We aimed to use this line for testing whether MNU treatment affected any of the *ubi:EGFP*-positive cone types. To strengthen this approach, we tested two monoclonal antibodies, Xap-2 and Xap-1, which were generated against the *Xenopus* retina (Harris and Messersmith, 1992). The Xap-2 antigen recognizes outer segments of rods in frogs and killifish (Choi et al., 2011; Berrosteguieta et al., 2022). The Xap-1 antigen has been mapped to Grp78 (Heat shock protein a5), which is detected in outer segments of rods and cones in frogs, but only of cones in mice, monkeys, and pigs (Nookala et al., 2010). The ability of the antibody to bind to photoreceptors has been correlated with proper outer segment formation in cones (Wohabrebbi et al., 2002). Interestingly, we found that both antibodies immunoreacted with the zebrafish retina, whereby the outer segment of UV cones was labeled by both markers (Figures 3E, F). In addition, Xap-2 immunodetected the outer segments of other photoreceptors with a similar intensity as that of UV cones, whereas Xap-1 displayed a dotty pattern that was much more diffuse in other photoreceptors. Double antibody staining with Xap-2 and Xap-1 suggests their colocalization in the outer segment of UV cones (Figure 4B).

Equipped with these new markers, we assessed morphology of *ubi:EGFP*-positive cones after MNU-treatment. At 5 dpMNU, *ubi:EGFP*^+^ short UV-cones were missing, and no Xap-2 and Xap-1 immunostaining was observed at the position normally corresponding to the outer segment of these cones (Figures 3E, F). Although retina morphology was disturbed, *ubi:EGFP*^+^/Xap-2^+^ blue cones were detected in MNU-treated retinas (Figures 3E, F). Similarly, ZPR-1-immunoreactive double red/green cones were also present, although their shape was disturbed, suggesting morphological abnormalities to double cones (Supplementary Figure 4C). We concluded that MNU injury not only damages rods, but also leads to degeneration of UV cones. This finding suggests that these two components of the outer nuclear layer are susceptible to this chemical treatment, triggering the regenerative program that can be monitored by *careg:EGFP*.

### Single-cell RNA sequencing of the regenerating retina after MNU-injury

**FIGURE 5:**
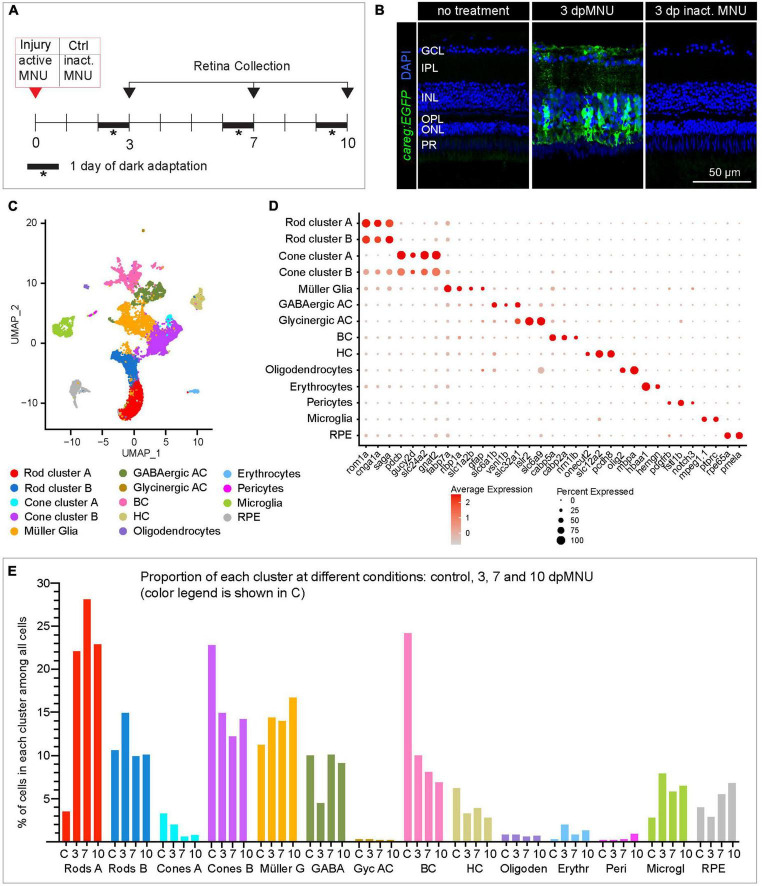
scRNA sequencing of *careg:EGFP* regenerating adult zebrafish retinas following MNU chemical injury. **(A)** Experimental design of retina isolation used for scRNA-sequencing. **(B)** Transversal sections of uninjured and regenerating *careg:EGFP* retinas at 3 days after treatment with harmful or inactivated MNU. *careg:EGFP* expression (green) is not detected after treatment with inactivated MNU, suggesting absence of injury. *N* = 3. **(C)** UMAP plots of the integrated cell RNA-sequencing data showing cluster assignments for each cell type collected from control and post-MNU treated retinas. **(D)** Dot plot showing expression of canonical markers for all retina cell types. Dot size indicates the proportion of cells expressing the corresponding gene and the color gradient indicates the average expression levels. **(E)** Histogram displaying percentage of cells in each cluster per time-point.

Our immunofluorescence analysis demonstrated that *careg:EGFP* is activated mainly in a subset of MG. To identify the molecular profile of these cells, we performed a scRNA-seq experiment with retinas dissected from adult *careg:EGFP* zebrafish. We selected three time-points at 3, 7, and 10 dpMNU, corresponding to the onset, the peak and the progressing-exit of the proliferative phase, respectively. Given that exposure to a chemical compound could on its own induce transcriptional changes, our control retinas were dissected from fish at 3 days after treatment with heat-inactivated MNU (Figure 5A). We confirmed that the administration of heat-inactivated MNU did not cause upregulation of *careg:EGFP* or injury, and thus, validating this control (Figure 5B).

Single-cell RNA sequencing analysis in Seurat pipeline included elimination of damaged or dying cells and doublets/triplets, which accounted for 12 ± 2% of all cells per condition (Supplementary Figure 5A). Following integration analysis of two replicates per condition, 17 distinct cell populations were identified, whose identity was annotated using known retina markers (Supplementary Table 2; Hoang et al., 2020; Ogawa and Corbo, 2021). Among them, we identified two subpopulations of GABAergic amacrine cells and three groups of bipolar cells, which we merged into one cluster per cell type, as they were beyond the focus of this study. The analysis captured only very few retinal ganglion cells, as none of the clusters was characterized by a unique *rbpms2b* expression. Interestingly, we identified two groups of rods and cones, each of which we maintained separately, as clusters A and B, respectively (Figures 5C, D). As the topic of our study on photoreceptor regeneration, we analyzed these subpopulations of each photoreceptor type.

Given that the composition of recovered cell types can be influenced by the status of cell adhesion and tissue integrity, we expected to obtain a higher yield of detached rods from MNU-treated retinas, which comprises a damaged nuclear layer. Indeed, the scRNA-seq data showed a much higher proportion of rods at 3, 7, and 10 dpMNU compared to uninjured retina. This result indicates a bias in cell survival and capture efficiency, as reported for other injury models of vertebrate retinas (Macosko et al., 2015; Clark et al., 2019; Hoang et al., 2020).

### Single-cell RNA sequencing revealed new markers of immature rods

First, we aimed to identify the distinctive features between the two rod clusters. In uninjured retinas, rod cluster A contained only 91 cells, corresponding to 3.5% of all cells, whereas regenerating retinas contained 6-times more cells in this cluster, namely, 22, 28, and 23% at 3, 7, and 10 dpMNU, respectively (Figure 5E, Supplementary Figures 5B, C, and Supplementary Table 3). This suggests that this population of rods expanded during regeneration.

**FIGURE 6:**
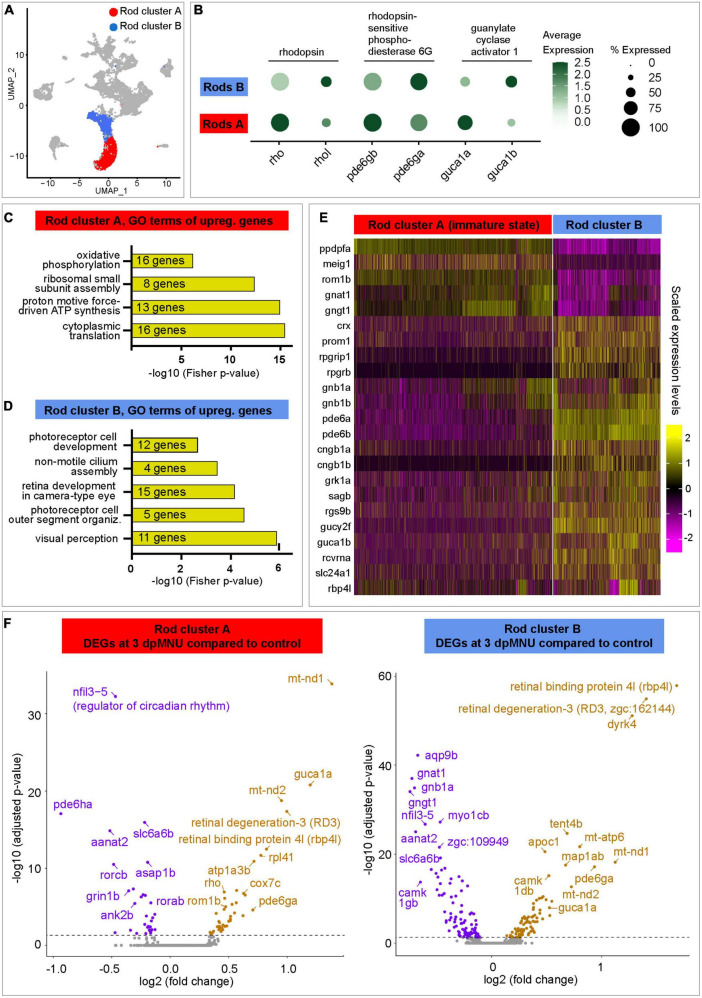
Transcriptome dynamics of immature and mature rods following MNU chemical injury. **(A)** UMAP plot of merged datasets showing the rod clusters in color and other cell clusters in gray. **(B)** Dot plot displaying differential expression levels of three pairs of paralogous genes of the rod identity in cluster A and B. **(C,D)** Selected Gene Ontology terms of upregulated genes in each rod cluster. Complete data are in Supplementary Table 4. **(E)** Heatmap of selected genes that display differential expression between both rod clusters. Complete data are in Supplementary Table 4. **(F)** Volcano plots of DEGs within each of rod clusters between 3 dpMNU compared to uninjured control. Complete data are in Supplementary Tables 5, 6.

Comparison between rod cluster A versus B reveled approximately 400 differentially expressed genes (DEGs) (Figure 6A and Supplementary Table 4). Interestingly, both subpopulations displayed an inversed enrichment of paralogous genes for three phototransduction factors, namely, *rhodopsin/rhodopsin-like (rho/rhol*), *phosphodiesterase 6 gamma paralog a/b (pde6ga/b*), and *guanylate cyclase activator 1 paralog a/b (guca1a/b*) (Figure 6B). To assess the functional difference between both cell populations, we performed Gene Ontology (GO) analysis of their DEGs. The cluster A comprised factors involved in translational processes and oxidative phosphorylation, suggesting increased cellular growth and metabolism (Figure 6C and Supplementary Table 4). Unlike cluster A, cluster B was characterized by genes linked to visual perception, photoreceptor cell outer segment organization, non-motile cilium assembly, photoreceptor cell development, and circadian gene expression, suggesting a mature status of rods in this group (Figure 6D). In cluster B, we identified several factors associated with retina diseases, such as retinitis pigmentosa (*rpgrip1* and *rpgrb*) and cone-rod dystrophy (*prom1* and *crx*) (Figure 6E and Supplementary Table 4). Importantly, cluster B displayed upregulated expression of the phototransduction pathway components, namely, *retinol-binding protein 4-like* (*rbp4l*), *recoverin a* (*rcvrna*), *G protein-coupled receptor kinase 1a* (*grk1a*), *rod arrestin* (*sagb*), *guanine nucleotide binding protein, beta polypeptide 1a/b* (*gnb1a/b*), *regulator of G protein signaling 9* (*rgs9b*), *phosphodiesterase 6a/b* (*pde6a/b*), *guanylate cyclase 2f* (*gucy2f*), *guanylate cyclase activator 1b* (*guca1b*), and *solute carrier family 24 member 1* (*slc24a1*) and *cyclic nucleotide gated channel subunit beta 1a/b* (*cngb1a/b*). Taken together, cluster A was different than cluster B, due to a lower expression of the visual perception genes, upregulation of translation genes, and a metabolic switch to oxidative phosphorylation. These characteristics suggest that cluster A contains immature rods.

Next, we aimed to better characterized the molecular signature of immature rods. Interestingly, two subunits of the transducin complex (*gnat1* and *gngt1*), were expressed at higher levels in immature than in mature rods (Figure 6E and Supplementary Table 4). In addition, a factor involved in the maintenance of the retina outer nuclear layer, *retinal outer segment membrane protein 1b* (*rom1b*), was also upregulated in immature rods. Among new factors, which have not been linked to retina functions, we identified *ppdpfa* (*pancreatic progenitor cell differentiation and proliferation factor a*) and *meig1* (*meiosis/spermiogenesis associated gene 1*), which are unique to this cluster (Supplementary Table 1). The latter gene is particularly interesting, because its mammalian ortholog is required for sperm axoneme assembly (Zhang et al., 2009). Given that various ciliated cells express *meig1*,^1^ and ciliogenesis is highly conserved among species (Avidor-Reiss and Leroux, 2015), we predict that *meig1* might be involved in the formation of the basal body/axoneme backbone during differentiation of the outer segment in zebrafish rod photoreceptors. This finding suggests that this axoneme-morphogenesis gene demarcates immature/maturating rods during assembly of the connecting cilium for the stabilization of the outer segment.

To uncover the molecular changes in photoreceptors of MNU-damaged retinas, we analyzed DEGs in immature and mature rod clusters between control and at different time point post-MNU treatment. In the immature rod cluster (rod cluster A), we found 74, 122, and 72 DEGs at 3, 7, and 10 dpMNU, respectively, compared to this cluster of uninjured retinas (Supplementary Table 5). In the cluster of mature rods (rod cluster B), we found 178, 148, and 182 DEGs at 3, 7 and 10 dpMNU, respectively (Supplementary Table 6). Within each cluster, many of DEGs were common for subsequent time-points after injury, suggesting persisting changes of the transcriptional profiles during regeneration. The most significant DEGs were graphically depicted in volcano plot for a comparison between 3 dpMNU versus control (Figure 6F). We noticed several common DEGs in both clusters after injury, such as regulators of circadian rhythm, *nfil3-5* and *aanat2*, which were downregulated in both clusters, whereas *retinal binding protein 4l* (*rbp4l*) and *retinal degeneration 3-like* (*rd3l, zgc:162144*) genes were upregulated. Furthermore, the genes highly expressed in cluster A, such as *pde6ga* and *guca1a*, as well as a several oxidative phosphorylation and metabolic genes were also upregulated in both rod clusters (Supplementary Tables 5, 6). Beside these similarities, several interesting differences were also observed between DEGs in both populations of rods. Specifically, mature rods (cluster B) downregulated *gnat1* and *gngt1*, the genes that are enriched in immature rods. On the other hand, immature rods (cluster A) upregulated their marker genes, such as *rho* and *rom1b*, in response to injury. Taken together, retina regeneration after MNU-injury is characterized by extensive transcriptomic changes of both immature and mature rods.

### Single-cell RNA sequencing revealed deregulation of cone function after MNU treatment

**FIGURE 7:**
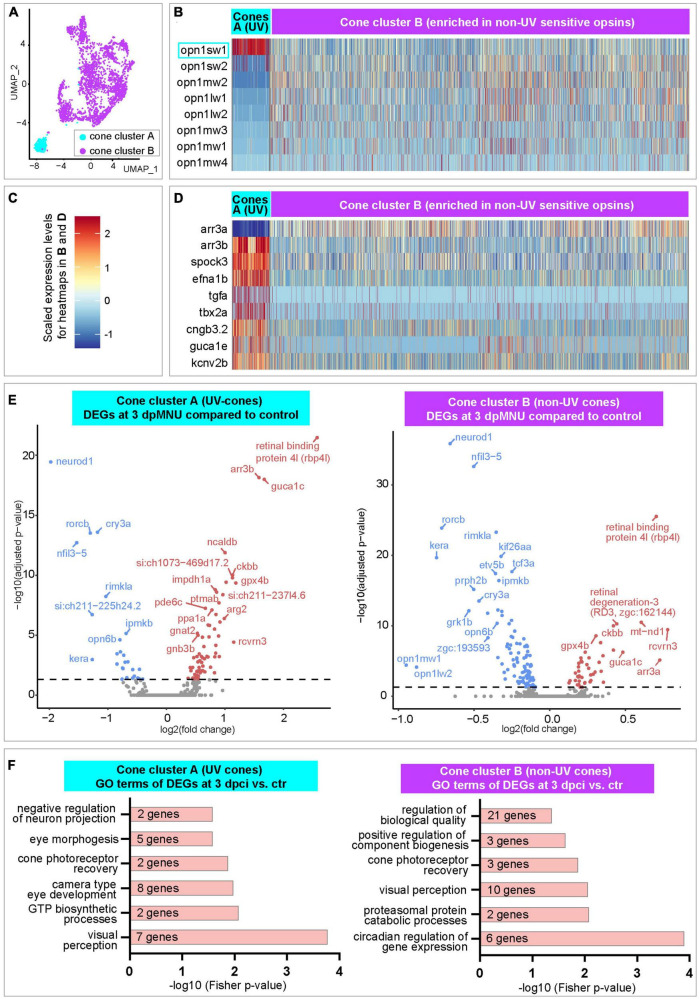
Transcriptome dynamics of UV and non-UV cones following MNU chemical injury. **(A)** UMAP plot of merged datasets showing the cone clusters in color and other cell clusters in gray. **(B)** Heatmap of *opsin-1* genes in cone clusters. Complete data are in Supplementary Table 7. **(C)** A three-color scale used to indicate expression levels in heatmaps in panels **(B,D)**. **(D)** Heatmap of selected genes in cone clusters. Complete data are in Supplementary Table 7. **(E)** Volcano plots of DEGs within each of cone clusters between 3 dpMNU compared to uninjured control. Complete data are in Supplementary Tables 8, 9. **(F)** Selected Gene Ontology terms of upregulated genes in each cone cluster. Complete data are in Supplementary Tables 8, 9.

Next, we extracted cone clusters A and B, and reanalyzed them to distinguish between UV and non-UV photoreceptors (Figure 7A). The cluster A demonstrated high expression of UV-sensitive *opn1sw1*, whereas cluster B displayed more expression of other visual *opsin-1* genes that are sensitive for non-UV spectra (Figures 7B, C and Supplementary Table 7). Both cone clusters were characterized by a nearly inverse abundance of *arrestin 3* (*arr3*) paralogous transcripts, whereby cluster A highly expressed *arr3b*, but was devoid of *arr3a* (Figures 7C, D and Supplementary Table 7). Among upregulated genes in cluster A as compared to B, we identified *spock3, efna1b, tgfa, tbx2a, cngb3.2 guca1e, kcnv2b*, all of which are associated with UV-light sensitivity (Ogawa and Corbo, 2021). We concluded that cluster A is enriched in UV-cones, whereas cluster B mostly contains non-UV cones.

Next, we analyzed dynamics of gene expression upon injury. In the UV-cone cluster A, we found 95, 18, and 68 DEGs at 3, 7, and 10 dpMNU, respectively, compared to uninjured retinas (Supplementary Table 8). In the cluster of non-UV cones (cluster B), we found 142, 217, and 348 DEGs at 3, 7, and 10 dpMNU, respectively (Supplementary Table 9). These numbers suggest that both clusters of cones were markedly affected in response to injury. Among the most significant DEGs, several genes were similarly deregulated in both clusters. At 3 dpMNU, common downregulated genes were *neurod1, opn6b* (a non-visual opsin), *rorcb, cry3a, nfil3-5, ipmkb, grk1b*, and *kera*, whereas common upregulated genes were *rbp4l, guca1c, rcvrn3, ckbb*, and cluster-specific *arr3* paralogs (Figure 7E and Supplementary Tables 8, 9). We have also identified many deregulated genes that are associated with visual, metabolic and cell survival processes (Figure 7F and Supplementary Tables 8, 9). These massive changes of transcriptome suggest that MNU treatment functionally impaired cone photoreceptors.

### *careg:EGFP*-positive cells express injury-induced genes in the Müller glia cluster

**FIGURE 8:**
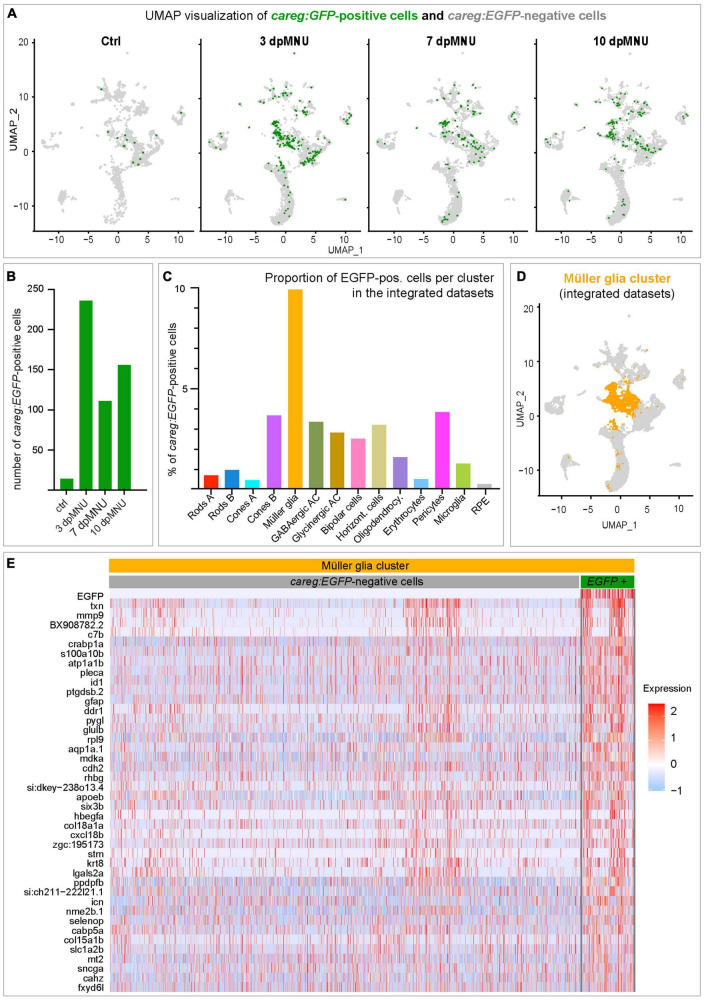
*careg:EGFP* is expressed in a subpopulation of Müller glia after MNU injury. **(A)** UMAP plots showing the distribution of *careg:EGFP*-positive cells (green) per condition. **(B)** Bar plot showing numbers of *careg:EGFP*-positive cells per time-point. **(C)** Histogram displaying the proportion of *careg:EGFP*-positive cells per cluster in the integrated cell RNA-sequencing data. **(D)** UMAP plot showing the distribution of the Müller glia cluster cells (orange) in the integrated scRNA-seq data. **(E)** A heatmap of differential gene expression analysis in *careg:EGFP*-positive cells compared to *EGFP*-negative cells within the Müller glia cluster. Complete data are in Supplementary Table 10.

In order to determine the molecular identity of *careg:EGFP*-expressing cells, we extracted all cells expressing *EGFP* transcripts, and we displayed them in UMAP plots (Figure 8A). While control retinas comprised only 14 *EGFP*-positive cells (0.54% of all cells), this number was 5- to 10-times higher in regenerating retinas (Figure 8B; 5.40% at 3 dpMNU, 2.64% at 7 dpMNU, and 2.66% at 10 dpMNU). This upregulation is consistent with our immunofluorescence analysis. Among all clusters, *EGFP*-positive cells predominantly mapped to MG, as EGFP transcripts were detected in nearly 10% of these cells, which is an outstanding proportion among other cell types (Figures 8C, D). Thus, we focused on this cluster for further analysis.

In the MG cluster, *careg:EGFP*-positive cells showed an enrichment for genes specific to this cell type, including *six3b*, *glulb, gfap*, *pleca, cahz*, *icn, txn*, and *apoeb* (Figure 8E and Supplementary Table 10). Consistent with regenerative activation, *careg:EGFP*-positive cells expressed higher levels of the proliferation-related gene *mdka*, and differentiation genes, such as *crabp1a, id1, ddr1*, and *lgals2a.* Furthermore, we also identified genes encoding extracellular matrix proteins, such as *stm, mmp9, hbegfa, col15a1b*, and *col18a1a*, and ion binding proteins, such as *fxyd6l, icn, sncga, mt2, selnop*, and *cabp5a*. Altogether, our analysis highlights a molecular distinction between *careg:EGFP*-negative and *careg:EGFP*-positive MG during retina regeneration in zebrafish.

### The activation of *careg* is independent of TOR signaling

**FIGURE 9:**
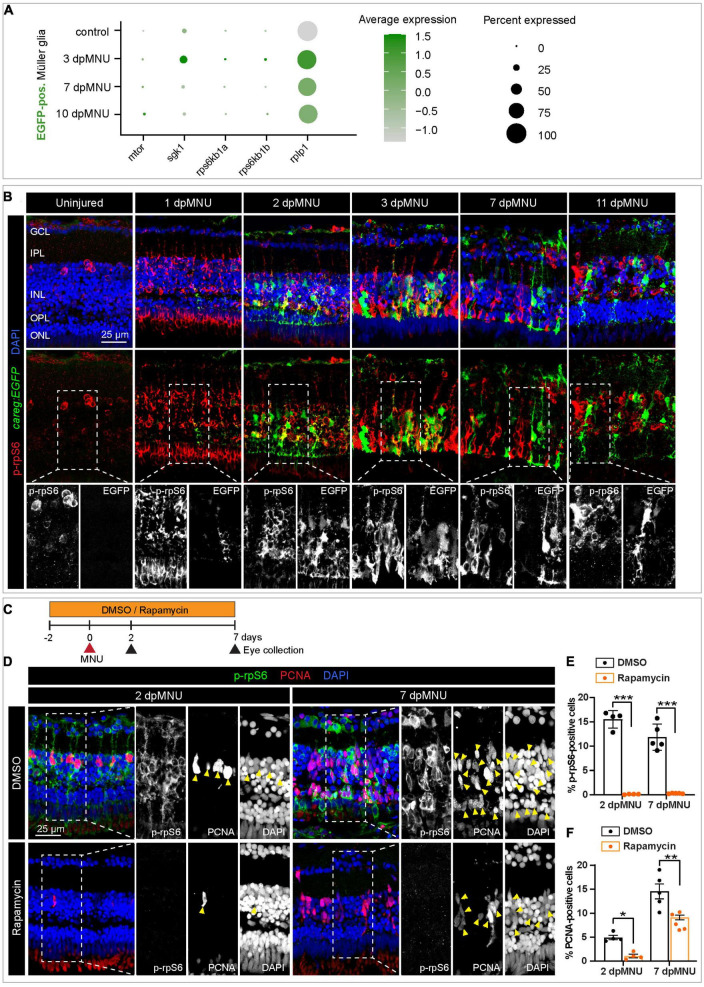
The *careg* element is transiently associated with phospho-rpS6-immunoreactive cells in regenerating retina. **(A)** Dot plot displaying differential expression levels of *mtor* and its downstream signaling components encoding ribosomal proteins in *careg:EGFP*-positive and *careg:EGFP*-negative Müller glia at different conditions. **(B)** *careg:EGFP* retina sections at different time-points post-MNU-treatment immunostained against phospho-ribosomal protein S6 (p-rpS6, red) and DAPI (blue). In uninjured retina, a few p-rpS6-positive cells (red) are detected in the inner nuclear layer (INL). At 1 dpMNU, a massive increase of p-rpS6 occurs across the INL. At 2 and 3 dpMNU, a colocalization between p-rpS6 (red) and *careg:EGFP* (green) is observed. At 7 and 11 dpMNU, most of cells are single-positive for each of these markers. **(C)** Workflow with 0.1% DMSO and 1 μM Rapamycin treatment. **(D)** Retina sections at 2 and 7 dpMNU treated with DMSO or Rapamycin immunostained for p-rpS6 (green), PCNA (red), and DAPI (blue). In control samples, PCNA-positive cells are also p-rpS6-positive (arrowheads). Rapamycin treatment abrogates p-rpS6 expression and reduced the number of PCNA-positive cells. **(E,F)** Quantification of PCNA-positive and p-rpS6-positive cells. Histogram displays average values for each group. Each dot represents a biological replicate (*N* > 4). Error bars, SEM. *P*-value was determined by two-way ANOVA with Šidák multiple comparisons test. *P = 0.034; **P = 0.0017; ***P < 0.001.

Recent studies have identified a relationship between the mammalian target of rapamycin complex 1 (mTORC1) pathway and photoreceptor survival in mice (Wang et al., 2022). The phosphorylated 40S ribosomal protein S6 (rpS6) is commonly used as a readout of mTORC1 activity in various contexts, including neuroscience (Biever et al., 2015). Inflammation-induced mTOR signaling is essential for retina regeneration in adult zebrafish after a stab injury (Zhang et al., 2020). To test whether this pathway is involved in the regulation of regenerative plasticity in the zebrafish retina after a non-invasive chemical injury, we compared the expression of selected relevant genes in our scRNA-seq analysis. Among the relevant DEGs, we identified *mtor*, ribosomal protein *rpS6*, and its kinases, *rpS6kb1a/b* in *careg:EGFP*-positive MG, suggesting the involvement of TOR signaling (Figure 9A). To address the hypothetical link between this pathway and the retinal response after MNU treatment, we assessed immunoreactivity against TOR regulation by phosphorylation of rpS6 (p-rpS6) in *careg:EGFP* regenerating retina. Consistent with a recent report on the needle-poke injury model (Zhang et al., 2020), we found that anti-rpS6 antibody was strongly induced at 1, 2, and 3 days post-MNU treatment. At this initial phase, in particularly at 2 dpMNU, we observed a colocalization between *careg:EGFP* and p-rpS6 (Figure 9B). However, at 7 and 11 dpMNU, this colocalization was no longer present, and p-rpS6 and *careg:EGFP*+ areas were not overlapping (Figure 9B). These results suggest that TOR signaling is only transiently associated with *careg:EGFP*-expressing MG shortly after injury, whereas both markers become uncoupled in proliferative progenitors.

**FIGURE 10:**
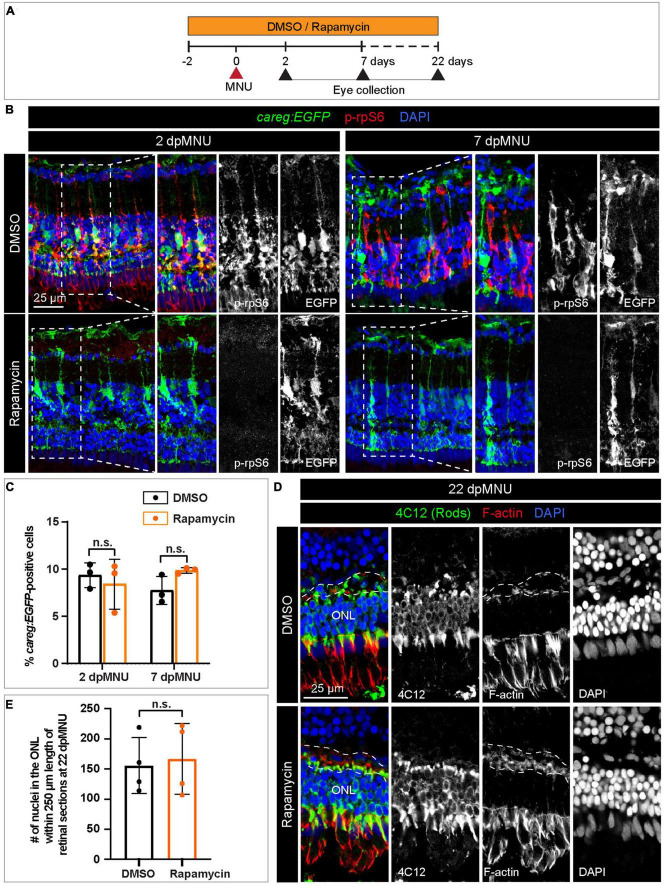
The *careg* element is not regulated by TOR signaling in regenerating retina. **(A)** Experimental design with 0.1% DMSO and 1 μM Rapamycin treatment. **(B)** Transversal sections of regenerating *careg:EGFP* (green) retinas at 2 and 7 dpMNU treated with DMSO or Rapamycin immunostained for the phosphorylated ribosomal protein p-rpS6 (red). Rapamycin treatment suppresses p-rpS6 immunoreactivity without affecting *careg:EGFP* expression (green). **(C)** Quantification of *careg:EGFP*-positive cells show a non-significant change between DMSO and Rapamycin-treated samples. Error bars, SEM. *P*-value was determined by two-way ANOVA with Sidák multiple comparisons test. n.s., not significant; *N* = 3. **(D)** Immunostaining of retina at 22 dpMNU demonstrates restoration of 4C12-positive rods (green) and F-actin-positive synaptic processes of the outer plexiform layer (encompassed with a dashed line). The position of outer nuclear layer (ONL) is indicated. **(E)** Quantification of nuclei in the outer nuclear layer within 250 μm length of retinal sections at 22 dpMNU. Error bars, SEM. *P*-value was determined by unpaired two-tailed Student’s *t*-test. n.s., not significant; *N* = 4.

To determine if *careg:EGFP* is regulated by TOR signaling, we inhibited this pathway with 1 μM Rapamycin (Figure 9C). We verified that this treatment completely abrogates p-rpS6 immunoreactivity, as shown at 2 and 7 dpMNU (Figures 9D, E). Using PCNA immunostaining, we found that this treatment also decreased cell proliferation (Figures 9D, F). Despite these effects, *careg:EGFP* expression remained unaffected (Figures 10A–C). Interestingly, at 22 dpMNU, immunostaining with rod marker 4C12 and Phalloidin revealed that the restoration of rods and photoreceptor synaptic processes was similar between DMSO-treated control and Rapamycin-treated retinas (Figures 10D, E). We concluded that TOR signaling is activated in MG to increase cell proliferation, but is not essential for *careg:EGFP* expression and the subsequent restoration of retina morphology in the MNU-induced injury model.

## Discussion

Mammalian photoreceptors are non-regenerative neuroepithelial cells, due to a lack of competent precursors in the retina. By contrast, zebrafish counterparts can be replaced by the activation of resident MG that give rise to proliferative progenitor cells (Bernardos et al., 2007; Thummel et al., 2010; Nagashima et al., 2013; Wan and Goldman, 2016; Gao et al., 2021). Current evidence suggests that the activation of cell plasticity in functional cells is orchestrated at the level of *cis*-regulatory sequences (Rodriguez and Kang, 2020). To tackle the molecular basis of this plasticity, we used a transgenic strain with the *careg* fluorescent reporter, which we have previously identified in the context of fin and heart regeneration (Pfefferli and Jaźwińska, 2017). We found that *careg:EGFP* was induced on the day after chemical injury and persisted for 2 months, up until completion of retina regeneration. Immunofluorescence analysis and scRNA analysis identified *careg:EGFP*-positive cells as a subpopulation of MG, highlighting a heterogeneity of this cell type during regeneration. Interestingly, *careg* was absent in MG and photoreceptors of zebrafish embryos and larvae, suggesting that this transgene is not responsive to retina developmental factors. This finding implies that regeneration involves distinct molecular bases to development, supporting previous models (Huang et al., 2012; Tanaka, 2016; Vieira et al., 2017). Thus, the *careg* reporter is selective for regeneration and not for uninjured progenitors and differentiating progeny at the circumferential germinal zone, in similarity with other reporters, such as *alpha1-tubulin:GFP* (Fausett and Goldman, 2006).

**FIGURE 11:**
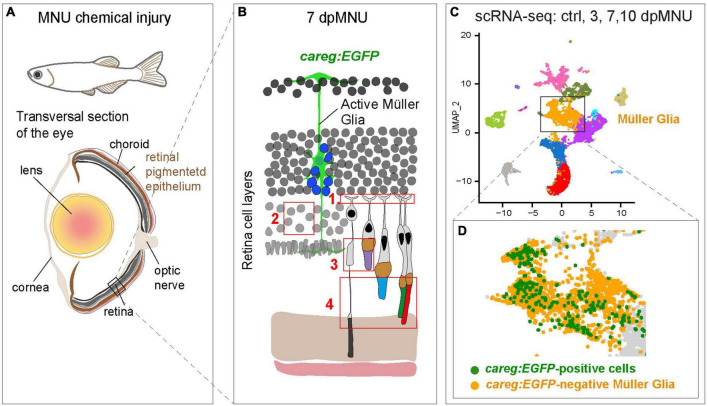
Identification of molecular differences between *careg:EGFP*-positive versus *careg:EGFP*-negative Müller glia during retina regeneration. **(A)** Schematic illustration of the experimental model in this study. **(B)** Cartoon of the retina at 7 dpMNU displays the induction of *careg:EGFP* expression in activated Müller glia (green cell), which give rise to the formation of proliferative progenitor cells (blue nuclei). The phenotypic defects of MNU-injury are highlighted in red frames: (1) Abolishment of actin filaments in synaptic photoreceptor processes in the outer plexiform layer. (2) Decrease of rod cell bodies in the outer nuclear layer. (3) Damage of inner segments of rod photoreceptors and the outer segments of UV-cones. (4) Distortion of photoreceptor outer segments. **(C)** UMAP visualization of cell clusters from the RNA-sequencing data of *careg:EGFP* retinas. **(D)** *careg:EGFP*-positive cells are detected mostly in the cluster of Müller glia.

We previously contributed to the establishment of the MNU-injury model, which impairs visual acuity and contrast sensitivity by disrupting the outer nuclear layer (Tappeiner et al., 2012, 2013). Histological analysis indicated disruption of rods. Here, we applied additional markers for fluorescent visualization of specific elements of photoreceptors. Our study expands the current knowledge by demonstration that MNU-injury leads not only to disorganization of rod cell bodies and photoreceptor inner segments, but also to damage of the outer plexiform layer, UV cones and outer segments of cones (Figures 11A, B). Thus, the consequences of MNU treatment could be more extensive than previously reported. Whether these aberrations are primary effects of MNU or a subsequent consequence of the overall disorganization of the retina requires further studies.

Using scRNA-seq analysis, we also identified the molecular signature of rods, cones, and MG at different time-points after injury (Figure 11C). We were able to identify several novel features concerning rods in the regenerating retina. At 3 days after damage, we found a 10-fold expansion of a new subpopulation of rods, which based on gene expression profile, might represent an immature state. Specifically, cells of this cluster display low expression of several genes of the photoreception pathway, as opposed to the second cluster of presumed mature rods. Interestingly, rhodopsin transcripts were more abundant in immature than mature rods, which highly expressed its *rhodopsin-like* paralog. A unique characteristic of immature rods is a distinctively high expression of axoneme morphogenesis gene, *meig1*. This gene has been identified as an essential factor for the sperm axoneme assembly in mice (Zhang et al., 2009). Like sperms, rod photoreceptors also rely on axoneme backbone in the connecting cilium that serves as a gate for trafficking of proteins and membrane components from the cell body to the outer segment (Nemet et al., 2015). Another markedly enriched gene was *ppdpfa*, which is a rarely investigated factor of differentiation and proliferation (Ma et al., 2021). Future follow-up studies are warranted to elucidate the expressional dynamics and the role of *meig1* and *ppdpfa* in zebrafish regenerating rods.

The results of the scRNA-seq allowed us to track cell identity and transcriptomic dynamics of *careg*-expressing cells. These cells were found mostly, although not exclusively, in the MG cluster (Figure 11D). The population of *careg:EGFP*-positive MG revealed a molecular signature with 42 upregulated genes, many of which were related to regeneration, actin filament organization and mitochondrial processes. Thus, the *careg:EGFP* element provides a tool to help understand the heterogeneity of MG cells during regeneration.

We found that translation genes and TOR signaling components are upregulated in *careg:EGFP*-positive MG after MNU treatment. We visualized the TOR pathway activity using phosphorylated-rpS6 immunostaining and inhibited the pathway with rapamycin. Consistent with a recent study (Zhang et al., 2020), p-rpS6 is extensively upregulated after MNU treatment and it enhances cell proliferation. Nevertheless, rapamycin treatment did not suppress *careg:EGFP* activation and the final restoration of photoreceptors in the MNU-injury model. This finding suggests that the activation of MG and the proliferative program of progenitor cells might be guided by independent mechanisms. The function of this pathway could be cell specific, given that the TOR activity is essential for retinal pigment epithelium regeneration in zebrafish (Lu et al., 2022). Further comparison of initial stimulation and subsequent proliferation is warranted to elucidate the molecular interactions that underlie photoreceptor restoration and the realignment of retinal layers.

## Conclusion

In summary, zebrafish provides a valuable model organism in neuroscience. The reversible induction of the *careg* reporter in regeneration-leading cells of the fin, the heart and the retina, provides unique evidence for the existence of common restorative biosensors across different organ types, including non-neural and neuronal tissues. Open questions remain as to whether other highly regenerative vertebrates, especially urodele amphibians, have evolved a comparable *cis*-regulatory element to guide restoration of their regeneration-competent cells.

## Data availability statement

The datasets presented in this study can be found in online repositories. The names of the repository/repositories and accession number(s) can be found in the article/Supplementary material. Single-cell RNA-seq data have been deposited to the Gene Expression Omnibus (GEO, accession number GSE202212).

## Ethics statement

The animal study was reviewed and approved by the Cantonal Veterinary Office in Fribourg.

## Author contributions

TB and CP carried out the lab work, performed the data analysis, designed the study, and drafted the manuscript. MB performed the lab work and data analysis. LT, HL, and RB performed the 10× analysis. AJ designed and coordinated the study and wrote the manuscript. All authors contributed to the article and approved the submitted version.

## Footnotes

1
proteinatlas.com
