# Supplementary material for: The regeneration-responsive element careg monitors activation of Müller glia after MNU-induced damage of photoreceptors in the zebrafish retina
Source: Front Mol Neurosci. 2023 Apr 17;16:1160707. doi: 10.3389/fnmol.2023.1160707 (PMC10149768; doi:10.3389/fnmol.2023.1160707)
Supplement: Supplementary file 2 [file Data_Sheet_2.pdf]

Supplementary Information

The regeneration-responsive *careg* element monitors activation of Müller glia after MNU-induced damage of photoreceptors in the zebrafish retina

Thomas Bise[1†], Catherine Pfefferli[1†], Marylène Bonvin[1], Lea Taylor[2,3], Heidi E.L. Lischer[2,3], Rémy Bruggmann[2,3] and Anna Jaźwińska[1*]

[1] *Department of Biology, University of Fribourg, Chemin du Musée 10, 1700 Fribourg, Switzerland*
[2] *Interfaculty Bioinformatic Unit, University of Bern, Baltzerstrasse 6, 3012 Bern, Switzerland*
[3] *Swiss Institute of Bioinformatics (SIB), 1015 Lausanne, Switzerland*

† equal contribution
* corresponding author: anna.jazwinska@unifr.ch

List of the Supplementary Material (not formatted for printing)

Supplementary Data 1
scRNAseq quality control report.

Supplementary Table 1
Identification of unique upregulated marker genes for each cluster.

Supplementary Table 2
Identification of clusters based on the known marker genes.

Supplementary Table 3
Cell numbers in each cluster per condition.

Supplementary Table 4
Transcriptomic comparison between rod cluster A versus rod cluster B.
3 sheets:
- Differentially expressed genes (DEGs) between rod cluster A and rod cluster B.
- Gene ontology (GO) terms of upregulated genes in rod cluster A.
- GO terms of upregulated genes in rod cluster B.

Supplementary Table 5
Analysis of rod cluster A (immature rods) at different time-points.
6 sheets
- DEGs in rod cluster A between 3 dpMNU versus control.
- DEGs in rod cluster A between 7 dpMNU versus control.
- DEGs in rod cluster A between 10 dpMNU versus control.
- GO terms of DEGs in rod cluster A between 3 dpMNU versus control.
- GO terms of DEGs in rod cluster A between 7 dpMNU versus control.
- GO terms of DEGs in rod cluster A between 10 dpMNU versus control.

Supplementary Table 6
Analysis of rod cluster B (mature rods) at different time-points.
6 sheets
- DEGs in rod cluster B between 3 dpMNU versus control.
- DEGs in rod cluster B between 7 dpMNU versus control.
- DEGs in rod cluster B between 10 dpMNU versus control.
- GO terms of DEGs in rod cluster B between 3 dpMNU versus control.
- GO terms of DEGs in rod cluster B between 7 dpMNU versus control.
- GO terms of DEGs in rod cluster B between 10 dpMNU versus control.

Supplementary Table 7
Transcriptomic comparison between cone cluster A versus cone cluster B.
3 sheets:
- Differentially expressed genes (DEGs) between cone cluster A and cone cluster B.
- GO terms of upregulated genes in cone cluster A (UV-cones).
- GO terms of upregulated genes in cone cluster B (non-UV-cones).

Supplementary Table 8
Analysis of cone cluster A (UV cones) at different time-points.
6 sheets
- DEGs in cone cluster A between 3 dpMNU versus control.
- DEGs in cone cluster A between 7 dpMNU versus control.
- DEGs in cone cluster A between 10 dpMNU versus control.
- GO terms of DEGs in cone cluster A between 3 dpMNU versus control.
- GO terms of DEGs in cone cluster A between 7 dpMNU versus control.
- GO terms of DEGs in cone cluster A between 10 dpMNU versus control.

Supplementary Table 9
Analysis of cone cluster B (non-UV cones) at different time-points.
6 sheets
- DEGs in cone cluster B between 3 dpMNU versus control.
- DEGs in cone cluster B between 7 dpMNU versus control.
- DEGs in cone cluster B between 10 dpMNU versus control.
- GO terms of DEGs in cone cluster B between 3 dpMNU versus control.
- GO terms of DEGs in cone cluster B between 7 dpMNU versus control.
- GO terms of DEGs in cone cluster B between 10 dpMNU versus control.

Supplementary Table 10
Analysis of Müller glia (MG) cluster.
DEGs between *careg:EGFP*-positive versus -negative cells in MG cluster.

Supplementary Figures 1 to 5 (in this document)

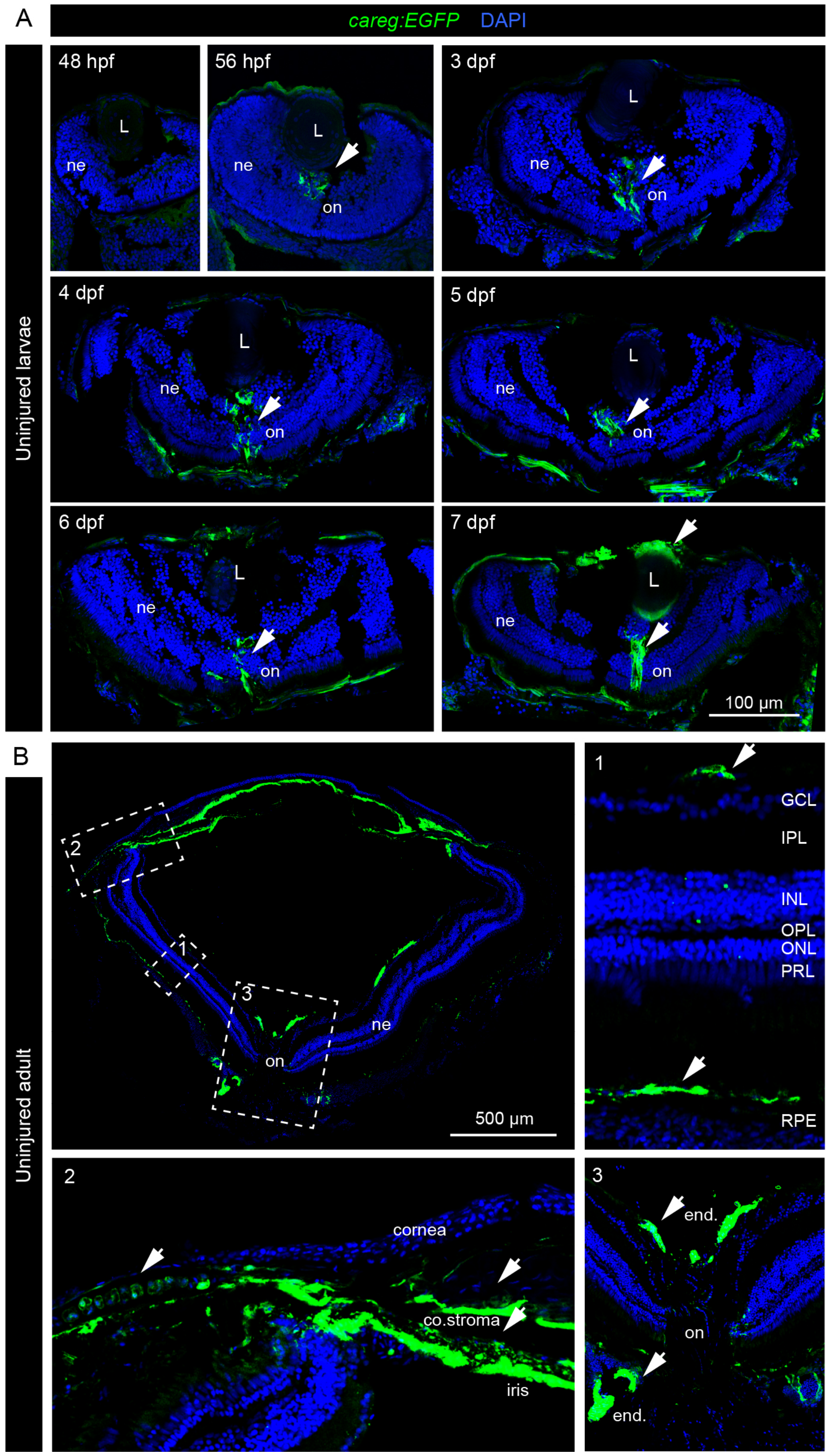

Supplementary Figure S1. ***careg:EGFP*** **expression in developing and adult uninjured zebrafish eye.**
(A) Transversal cryosections of *careg:EGFP* transgenic larval eyes at different developmental stages. Endogenous *careg:EGFP* expression is shown in green (white arrows) and nuclei are stained with DAPI (blue). Number of fish, N = 3.
(B) Transversal cryosections of *careg:EGFP* adult eyes. Higher magnifications of the adult eye are shown as 1-3 panels. 1. Retina. 2. Corneal stroma and iris. 3. Region with the optic nerve. L, lens; ne, neuroepithelium; on, optic nerve; co.stroma, corneal stroma; end., endothelium.

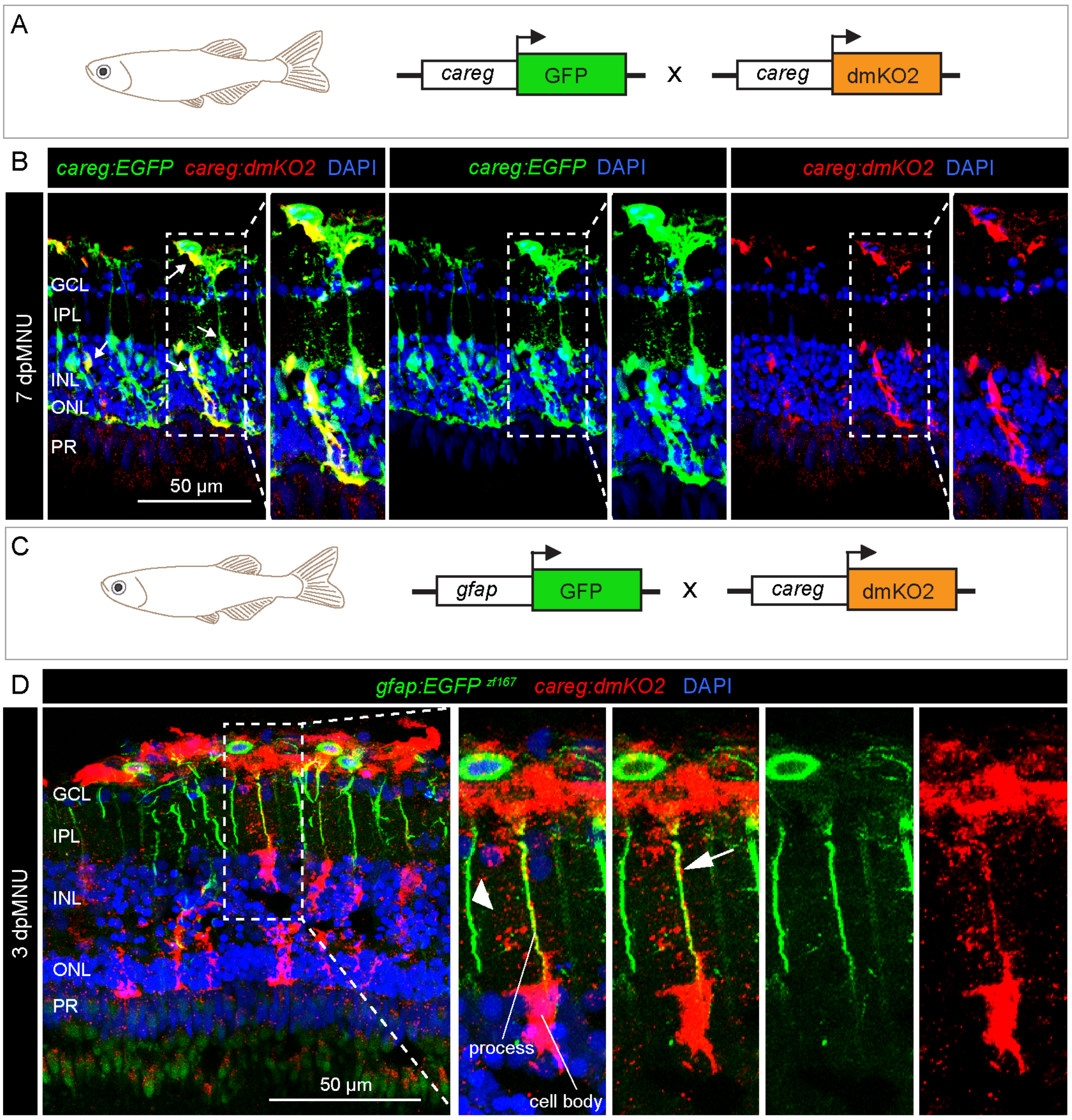

Supplementary Figure S2. Expression of *careg:dmKO2* during retina regeneration.
(A) Schematic representation of the double transgenic *careg:EGFP;careg:dmKO2* zebrafish.
(B) Retina section at 7 dpMNU demonstrates that both transgenic reporters partially overlap in elongated cells of the retina
(C) Schematic representation of the double transgenic *gfap:EGFP*^{zf167}*;careg:dmKO2* zebrafish.
(D) *gfap:EGFP*^{zf167} demarcates the processes of Müller glia, some of which are also *careg:dmKO2*-positive.
GCL, ganglion cell layer; IPL, inner plexiform layer; INL, inner nuclear layer; OPL, outer plexiform layer; ONL, outer nuclear layer; PRL, photoreceptors layer. N=3.

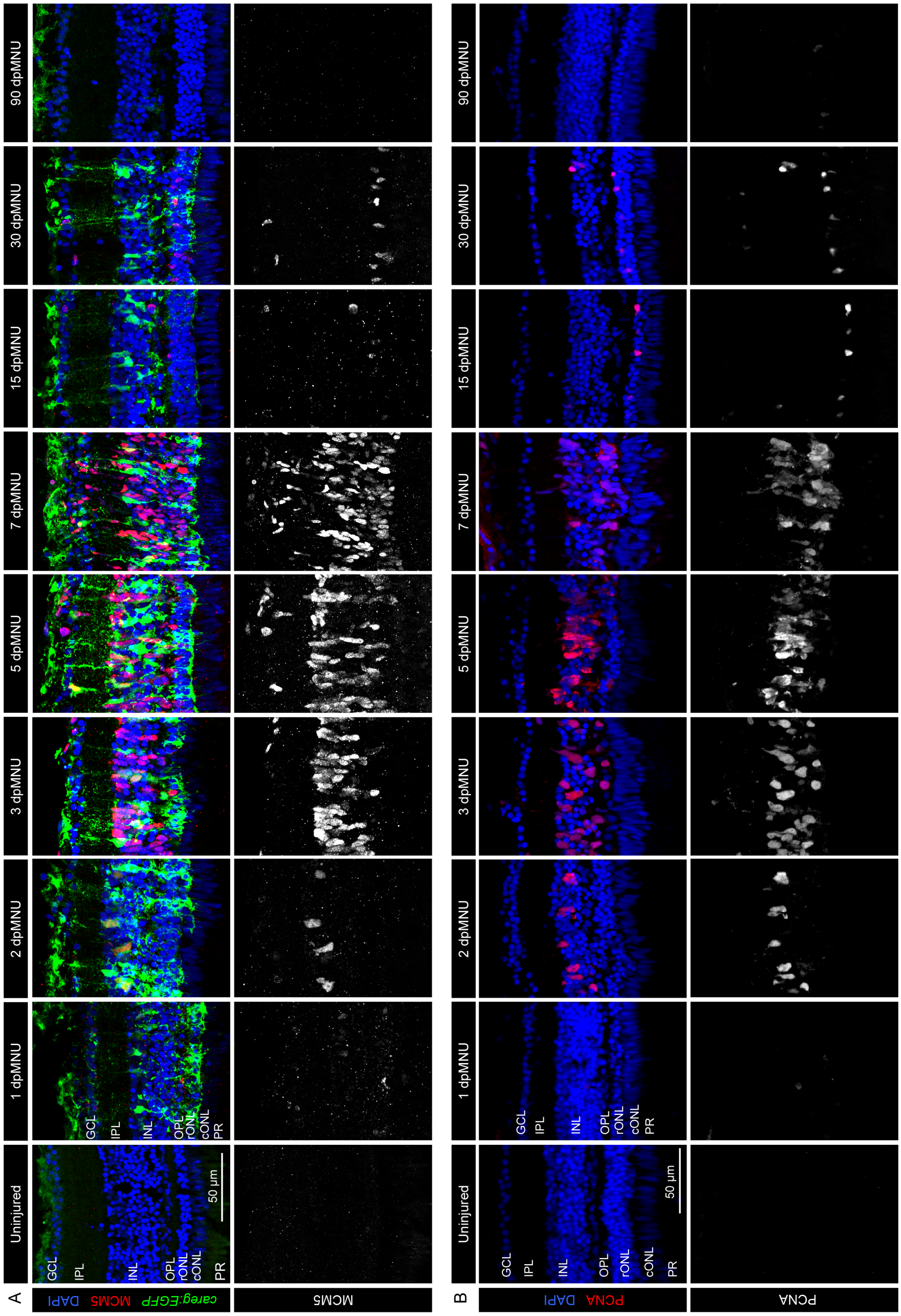

Supplementary Figure S3. The distribution of MCM5 and PCNA expressing cells is similar during retina regeneration.

(A) Sections of *careg:EGFP* retinas immunostained against MCM5 (red), without antigen retrieval.

(B) Section immunostained against PCNA following antigen retrieval. N ≥ 3.

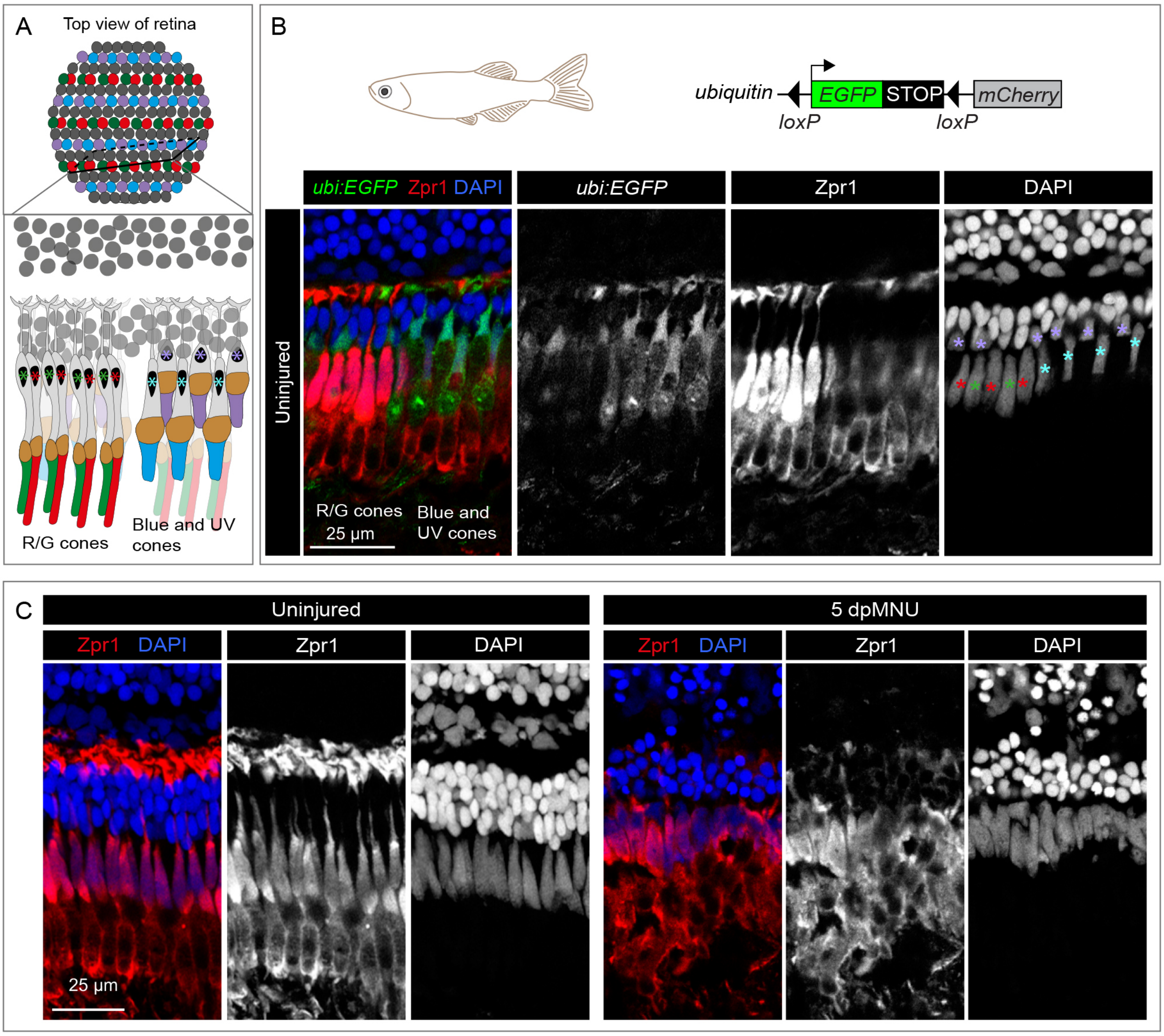

Supplementary Figure S4. The outer segments of red/green R/G cones are disorganized after MNU treatment.

(A) Schematic explanation of the retina sections that include areas with R/G cones and UV and blue cones. Asterisks demarcate the nuclei of different cones with the color code.

(B) Immunostaining against R/G cone marker Zpr1 (red) of uninjured retina of ubi:EGFP (green) transgenic fish. The area with intense Zpr1 staining does not colocalize with ubi:EGFP, which is specific for UV and blue cones. N=3.

(C) Immunostaining against R/G cone marker Zpr1 (red) of uninjured and 5 dpMNU retinas. Disorganized pattern of Zpr1-positive outer segment of R/G cones is detected at 5 dpMNU. N=3.

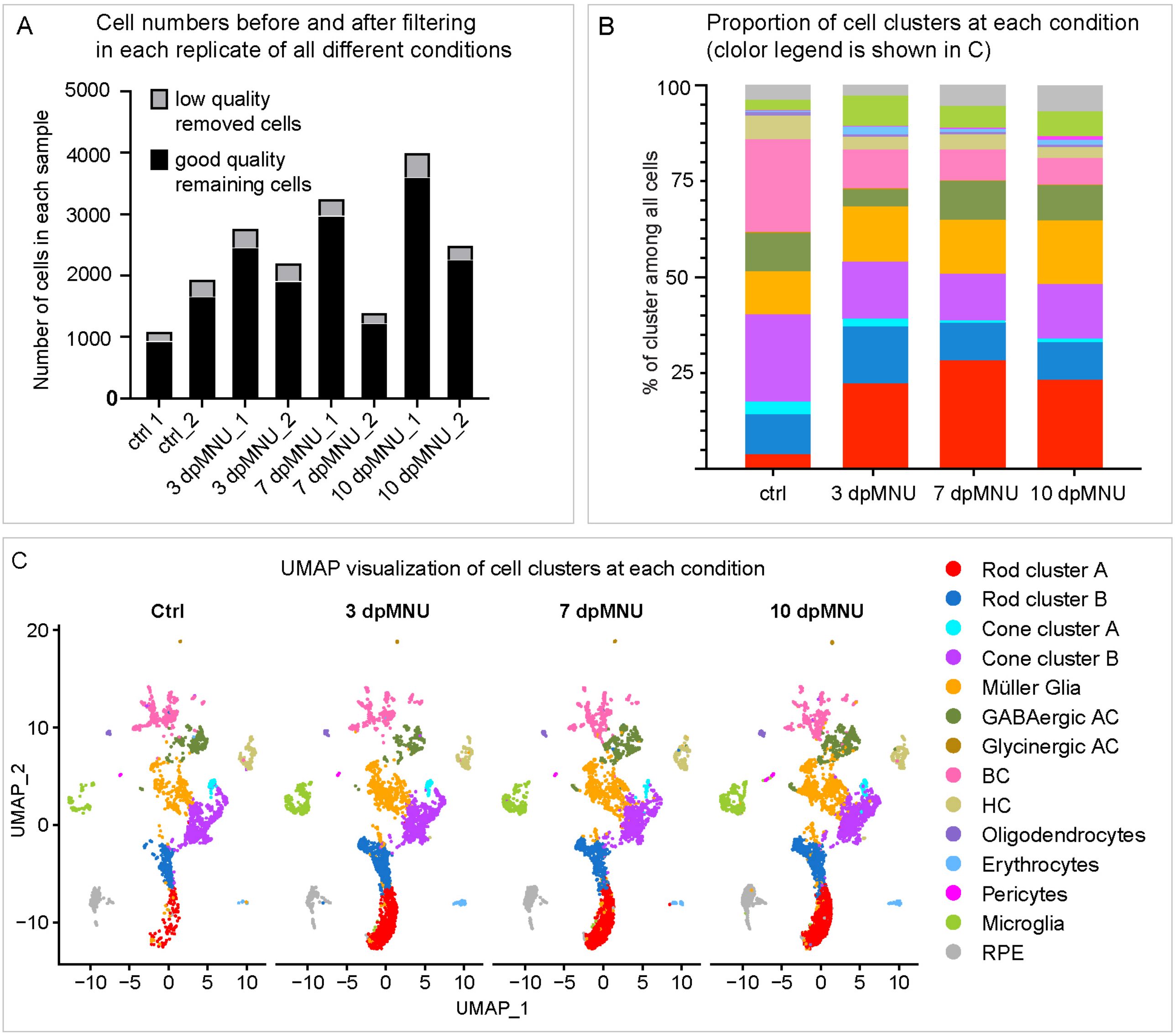

Supplementary Figure S5. Cell composition of the adult zebrafish retina after MNU treatment.
(A) Graphical representation of the cell numbers recovered in each biological replicate of scRNAseq.
(B) Proportion of the cell clusters for control uninjured retinas and for the three timepoints following MNU chemical injury of the scRNA-sequencing data.
(C) UMAP visualization of cell clusters for control uninjured retinas and for the three timepoints following MNU chemical injury of the scRNA-sequencing data.
