# Supplementary material for: The regeneration-responsive element careg monitors activation of Müller glia after MNU-induced damage of photoreceptors in the zebrafish retina
Source: Front Mol Neurosci. 2023 Apr 17;16:1160707. doi: 10.3389/fnmol.2023.1160707 (PMC10149768; doi:10.3389/fnmol.2023.1160707)
Supplement: Supplementary file 1 [file Data_Sheet_1.PDF]

1. Title and authorship of the publication
2. Quality control
3. Filtering
4. Normalization
Session Information

The regeneration-responsive careg element monitors activation of Müller glia after MNU-induced damage of photoreceptors in the zebrafish retina

Thomas Bise[1†], Catherine Pfefferli[1†], Marylène Bonvin[1], Lea Taylor[2,3], Heidi E.L. Lischer[2,3] Rémy Bruggmann[2,3] and Anna Jaźwińska[1*]

1 Department of Biology, University of Fribourg, Chemin du Musée 10, 1700 Fribourg, Switzerland
2 Interfaculty Bioinformatic Unit, University of Bern, Baltzerstrasse 6, 3012 Bern, Switzerland
3 Swiss Institute of Bioinformatics (SIB), 1015 Lausanne, Switzerland

† equal contribution
* corresponding author: anna.jazwinska@unifr.ch

# scRNAseq_qc-filtering-normalization

David Francisco
Interfaculty Bioinformatics Unit (IBU)
david.francisco@bioinformatics.unibe.ch

2. Quality control

Tool: Scater

Library size versus detected genes

A high number of detected genes can potentially indicate doublets. However, depending on the celltype composition in your sample, it may also reflect true biological variation among cell types.

- sum: total number of counts for a cell (i.e. library size)
- detected: total number of genes expressed in a cell

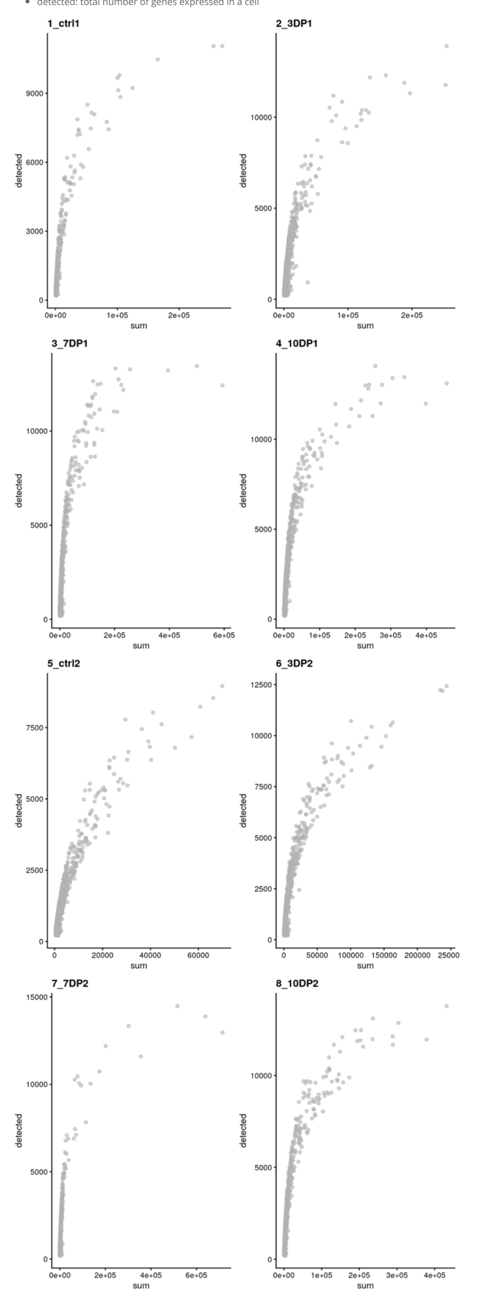

Percent of mitochondrial reads

Low quality /dying cells often exhibit extensive mitochondrial contamination. Cells with a high proportion of mitochondrial reads will be removed.

- detected: total number of genes expressed in a cell
- subsets_Mito_percent: percentage of all counts that come from the mitochondrial genome

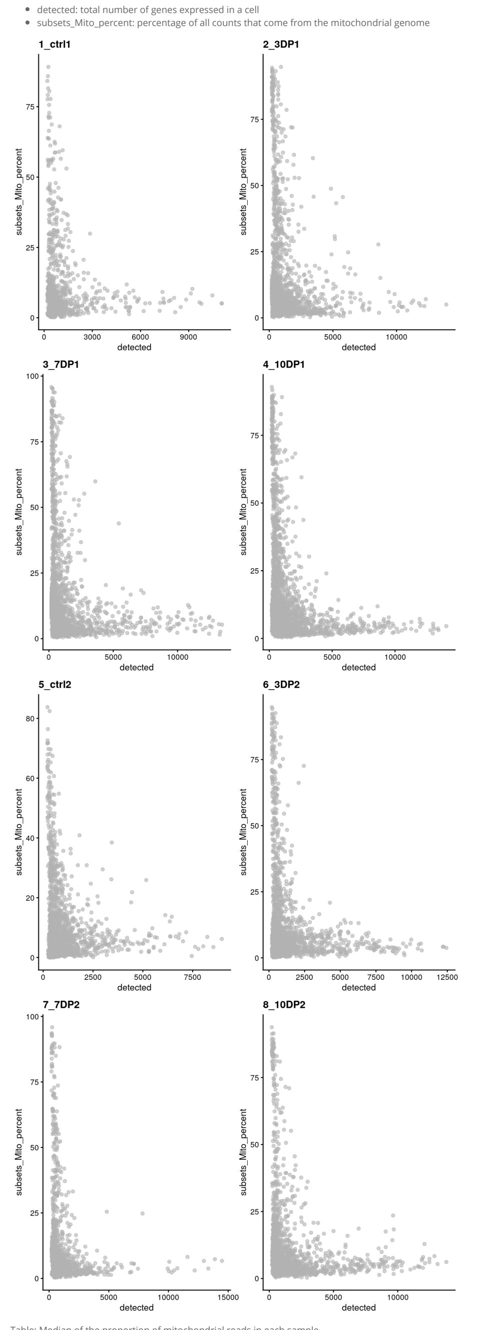

Table: Median of the proportion of mitochondrial reads in each sample

	median [%]
1_ctrl1	5.740149
2_3DP1	8.520179
3_7DP1	9.893455
4_10DP1	7.917889
5_ctrl2	4.918507
6_3DP2	5.740741
7_7DP2	6.106049
8_10DP2	6.617647

Percent of ribosomal protein reads

The fraction of reads from ribosomal proteins varies based on cell type and overall cell health. Higher levels of RNA degradation may lead to more templating of ribosomal proteins.

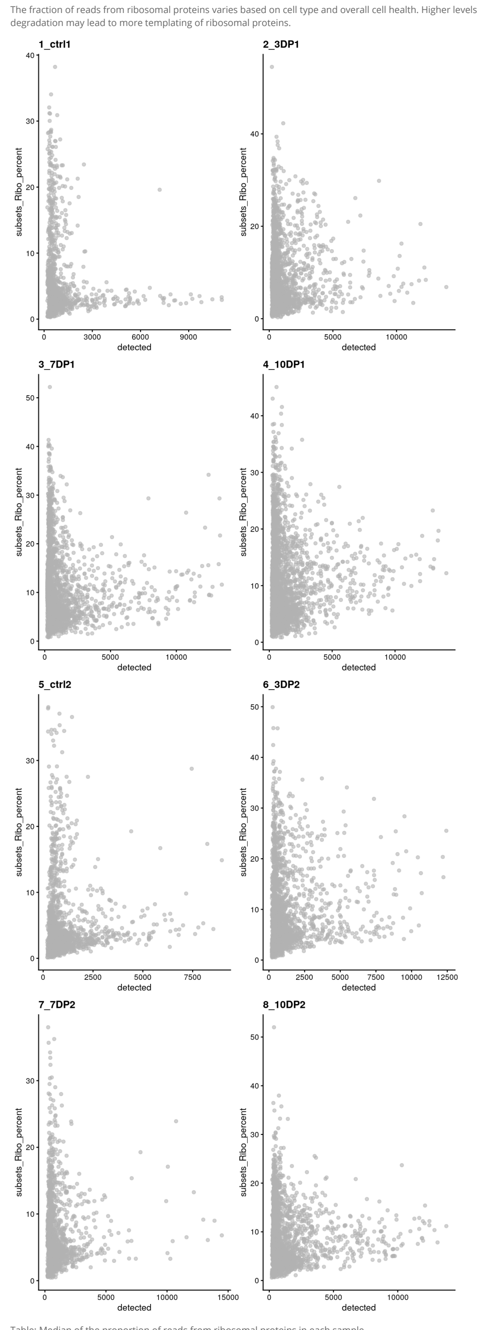

Table: Median of the proportion of reads from ribosomal proteins in each sample

	median [%]
1_ctrl1	3.711002
2_3DP1	7.479675
3_7DP1	9.545454
4_10DP1	8.650588
5_ctrl2	2.832758
6_3DP2	6.717134
7_7DP2	5.268020
8_10DP2	6.625644

3. Filtering

Tool: Scater

Automatically identifying low-quality cells

Scater provides an approach to automatically remove low-quality cells based on experiment-specific aspects of the data.

Table: Number of cells removed due to low library size (**low_lib_size**), low number of expressed genes (**low_n_features**), high proportion of mitochondrial reads (**high_subset_Mito_percent**) and in total (**discard**) in each sample.

	low_lib_size	low_n_features	high_subsets_Mito_percent	discard
1_ctrl1	0	0	160	160
2_3DP1	0	0	320	320
3_7DP1	0	0	281	281
4_10DP1	0	0	412	412
5_ctrl2	0	0	284	284
6_3DP2	0	0	301	301
7_7DP2	0	0	179	179
8_10DP2	0	0	234	234

Gene-level QC

Plots showing the top 50 most highly expressed genes in each sample. Each row corresponds to a gene, and each boxplot corresponds to the expression of a gene (i.e. number of reads) in a single cell. The vertical line in within the box indicates the median expression of each gene across all cells. Genes are sorted in decreasing order based on median expression.

Sometimes individual genes may have very high expression and should be removed to avoid problems at the normalization step. In particular, look out for MALAT1 and other nuclear lincRNAs, mitochondrial genes (prefix mt-), ribosomal proteins (starting with rp), actin and hemoglobin.

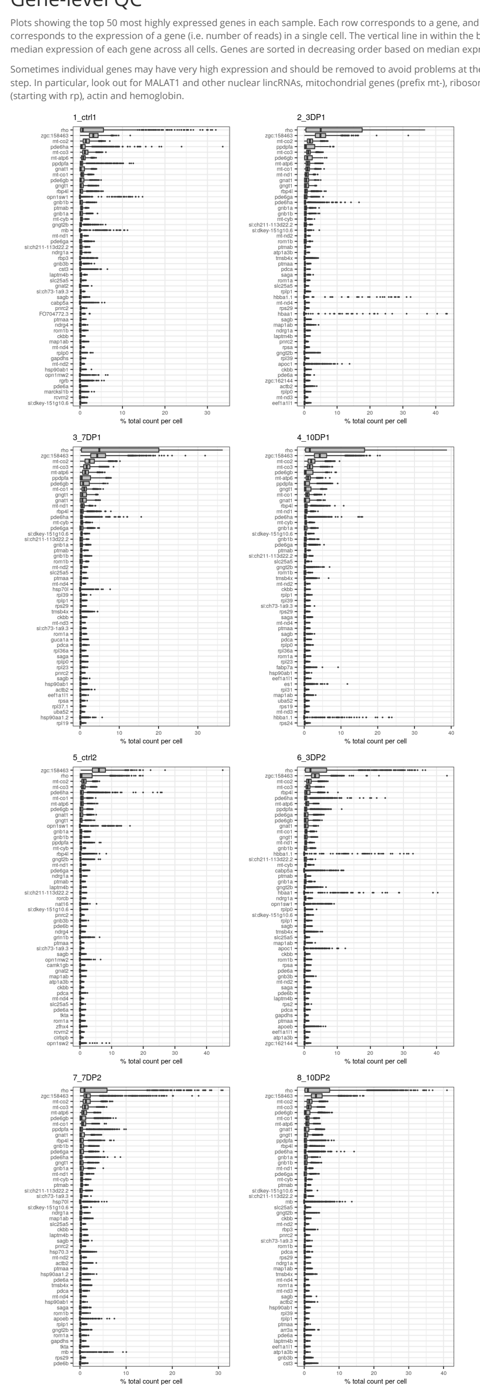

Statistics after filtering

Number of cells

	Before filtering	After filtering
1_ctrl1	1096	936
2_3DP1	2777	2457
3_7DP1	3257	2976
4_10DP1	4015	3603
5_ctrl2	1946	1662
6_3DP2	2216	1915
7_7DP2	1410	1231
8_10DP2	2500	2266

Library size versus detected genes after filtering

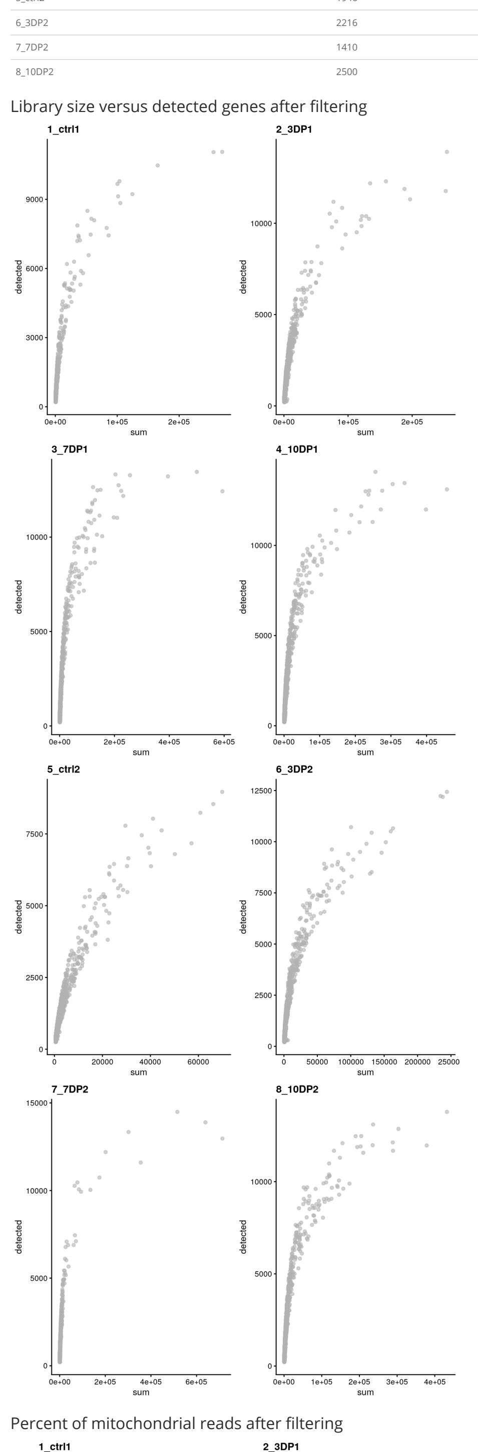

Percent of mitochondrial reads after filtering

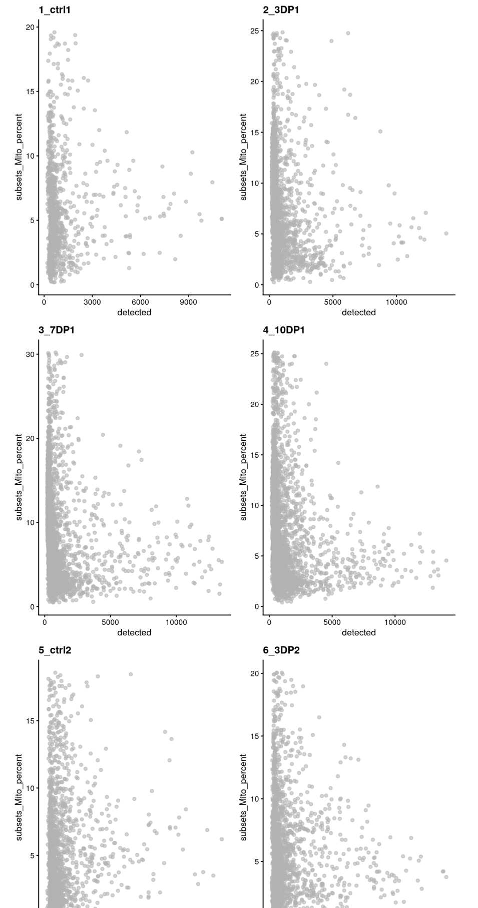

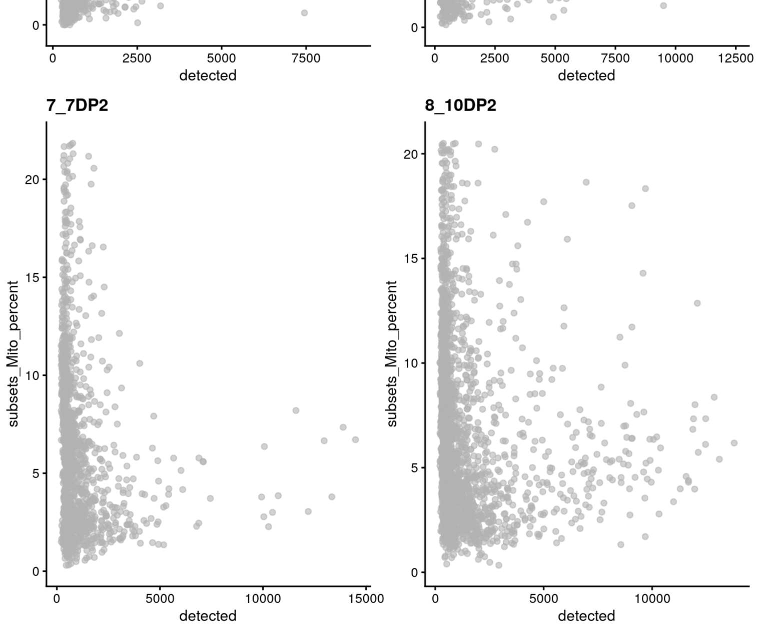

Percent of ribosomal protein reads after filtering

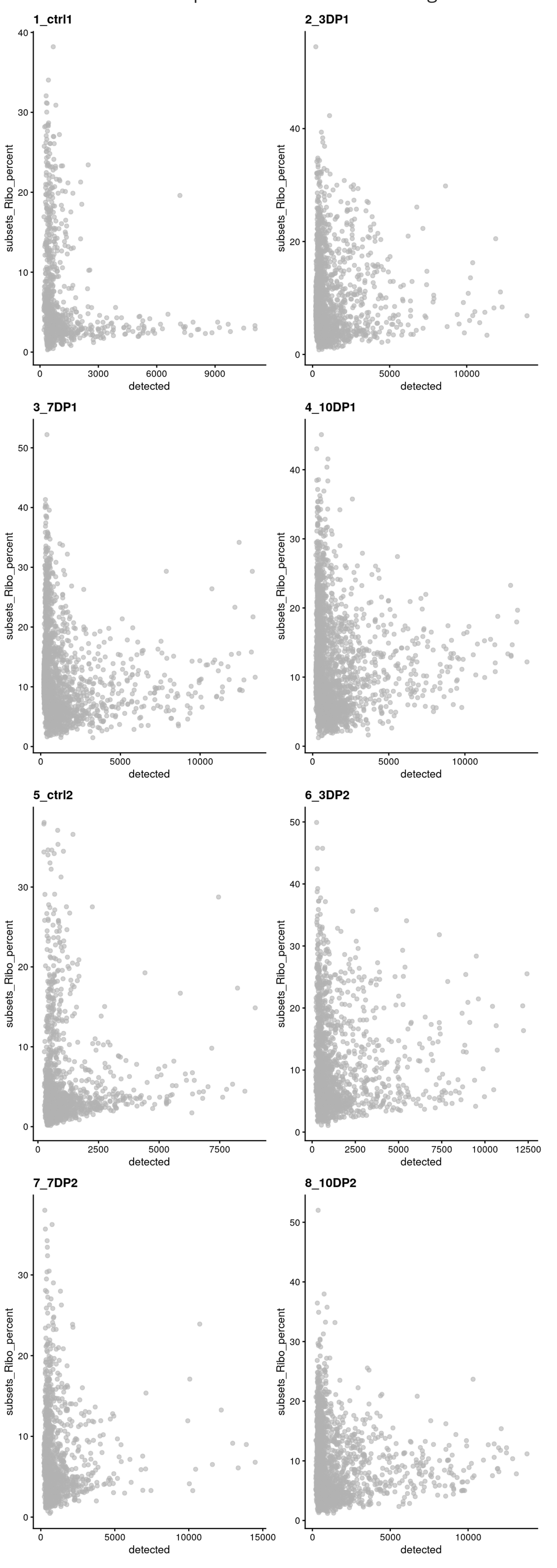

4. Normalization

Tool: Seurat

Normalization is applied to correct for differences in library size between samples. Biological heterogeneity in single-cell RNA-seq data is often confounded by technical factors including sequencing depth. The number of molecules detected in each cell can vary significantly between cells, even within the same celltype.

Therefore, we apply sctransform normalization (Hafemeister and Satija, Genome Biology 2019), which builds regularized negative binomial models of gene expression in order to account for technical artifacts while preserving biological variance. During the normalization, we also remove confounding sources of variation (mitochondrial and ribosomal mapping percentage).

before normalization:

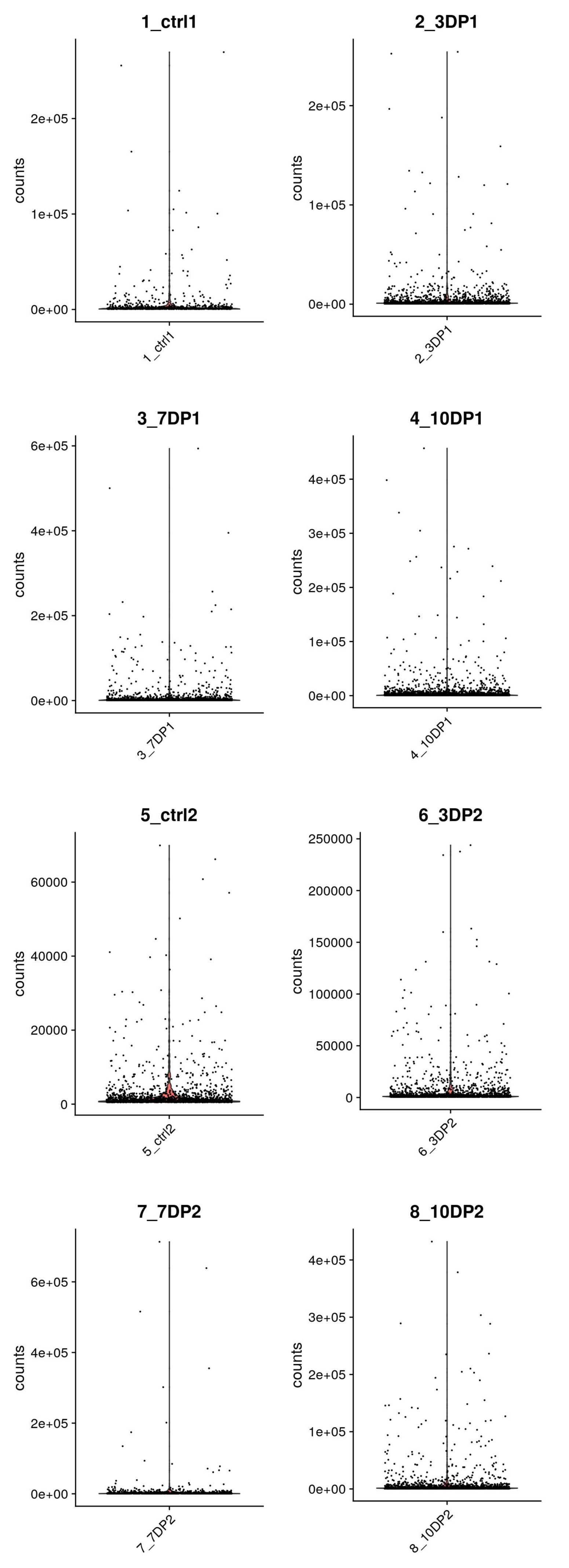

after normalization:

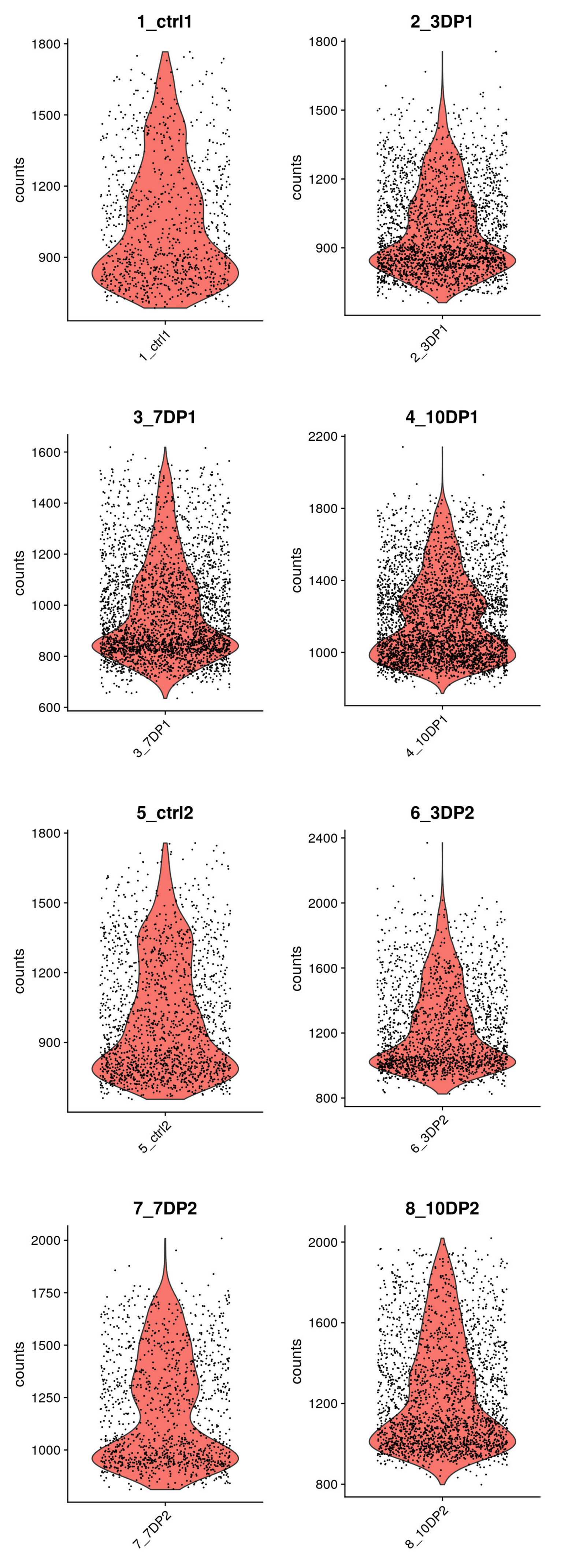

Session Information

R version 4.1.0 (2021-05-18) Platform: x86_64-pc-linux-gnu (64-bit) Running under: Debian GNU/Linux bullseye/sid

Matrix products: default BLAS: /usr/lib/x86_64-linux-gnu/openblas-pthread/libblas.so.3 LAPACK: /usr/lib/x86_64-linux-gnu/openblas-pthread/libopenblasp-r0.3.13.so

locale: [1] LC_CTYPE=en_US.UTF-8 LC_NUMERIC=C
[3] LC_TIME=en_US.UTF-8 LC_COLLATE=en_US.UTF-8
[5] LC_MONETARY=en_US.UTF-8 LC_MESSAGES=en_US.UTF-8
[7] LC_PAPER=en_US.UTF-8 LC_NAME=C
[9] LC_ADDRESS=C LC_TELEPHONE=C
[11] LC_MEASUREMENT=en_US.UTF-8 LC_IDENTIFICATION=C

attached base packages: [1] stats4 stats graphics grDevices utils datasets methods
[8] base

other attached packages: [1] knitr_1.39 ggpubr_0.4.0
[3] kableExtra_1.3.4 scran_1.22.1
[5] umap_0.2.8.0 scater_1.22.0
[7] ggplot2_3.3.6 scuttle_1.4.0
[9] SingleCellExperiment_1.16.0 SummarizedExperiment_1.24.0 [11] Biobase_2.54.0 GenomicRanges_1.46.1
[13] GenomeInfoDb_1.30.1 IRanges_2.28.0
[15] S4Vectors_0.32.4 BiocGenerics_0.40.0
[17] MatrixGenerics_1.6.0 matrixStats_0.62.0
[19] sp_1.5-0 SeuratObject_4.1.0
[21] Seurat_4.1.1

loaded via a namespace (and not attached): [1] utf8_1.2.2 reticulate_1.25
[3] tidyselect_1.1.2 htmlwidgets_1.5.4
[5] grid_4.1.0 BiocParallel_1.28.3
[7] Rtsne_0.16 munsell_0.5.0
[9] ScaledMatrix_1.2.0 codetools_0.2-18
[11] ica_1.0-3 statmod_1.4.36
[13] future_1.27.0 miniUI_0.1.1.1
[15] withr_2.5.0 spatstat.random_2.2-0
[17] colorspace_2.0-3 progressr_0.10.1
[19] highr_0.9 rstudioapi_0.13
[21] ROCR_1.0-11 ggsignif_0.6.3
[23] tensor_1.5 listenv_0.8.0
[25] labeling_0.4.2 GenomeInfoDbData_1.2.7
[27] polyclip_1.10-0 farver_2.1.1
[29] parallelly_1.32.1 vctrs_0.4.1
[31] generics_0.1.3 xfun_0.31
[33] R6_2.5.1 ggbeeswarm_0.6.0
[35] rsvd_1.0.5 locfit_1.5-9.5
[37] bitops_1.0-7 spatstat.utils_2.3-1
[39] cachem_1.0.6 DelayedArray_0.20.0
[41] assertthat_0.2.1 promises_1.2.0.1
[43] scales_1.2.0 rgeos_0.5-9
[45] beeswarm_0.4.0 gtable_0.3.0
[47] beachmat_2.10.0 globals_0.15.1
[49] goftest_1.2-3 rlang_1.0.4
[51] systemfonts_1.0.4 splines_4.1.0
[53] rstatix_0.7.0 lazyeval_0.2.2
[55] broom_1.0.0 spatstat.geom_2.4-0
[57] yaml_2.3.5 reshape2_1.4.4
[59] abind_1.4-5 backports_1.4.1
[61] httpuv_1.6.5 tools_4.1.0
[63] ellipsis_0.3.2 spatstat.core_2.4-4
[65] jquerylib_0.1.4 RColorBrewer_1.1-3
[67] ggridges_0.5.3 Rcpp_1.0.9
[69] plyr_1.8.7 sparseMatrixStats_1.6.0
[71] zlibbioc_1.40.0 purrr_0.3.4
[73] RCurl_1.98-1.7 rpart_4.1.16
[75] openssl_2.0.2 deldir_1.0-6
[77] pbapply_1.5-0 viridis_0.6.2
[79] cowplot_1.1.1 zoo_1.8-10
[81] ggrepel_0.9.1 cluster_2.1.3
[83] magrittr_2.0.3 data.table_1.14.2
[85] RSpectra_0.16-1 scattermore_0.8
[87] lmtest_0.9-40 RANN_2.6.1
[89] fitdistrplus_1.1-8 patchwork_1.1.1
[91] mime_0.12 evaluate_0.15
[93] xtable_1.8-4 gridExtra_2.3
[95] compiler_4.1.0 tibble_3.1.8
[97] KernSmooth_2.23-20 htmltools_0.5.3
[99] mgcv_1.8-40 later_1.3.0
[101] tidyr_1.2.0 DBI_1.1.3
[103] MASS_7.3-57 car_3.1-0
[105] Matrix_1.4-1 cli_3.3.0
[107] parallel_4.1.0 metapod_1.2.0
[109] igraph_1.3.4 pkgconfig_2.0.3
[111] plotly_4.10.0 spatstat.sparse_2.1-1
[113] xml2_1.3.3 svglite_2.1.0
[115] vipor_0.4.5 bslib_0.4.0
[117] dqrng_0.3.0 webshot_0.5.3
[119] XVector_0.34.0 rvest_1.0.2
[121] stringr_1.4.0 digest_0.6.29
[123] sctransform_0.3.3 RcppAnnoy_0.0.19
[125] spatstat.data_2.2-0 rmarkdown_2.14
[127] leiden_0.4.2 uwot_0.1.11
[129] edgeR_3.36.0 DelayedMatrixStats_1.16.0 [131] shiny_1.7.2 lifecycle_1.0.1
[133] nlme_3.1-158 jsonlite_1.8.0
[135] carData_3.0-5 BiocNeighbors_1.12.0
[137] viridisLite_0.4.0 askpass_1.1
[139] limma_3.50.3 fansi_1.0.3
[141] pillar_1.8.0 lattice_0.20-45
[143] fastmap_1.1.0 httr_1.4.3
[145] survival_3.3-1 glue_1.6.2
[147] png_0.1-7 bluster_1.4.0
[149] stringi_1.7.8 sass_0.4.2
[151] BiocSingular_1.10.0 dplyr_1.0.9
[153] irlba_2.3.5 future.apply_1.9.0
